# Targeting NLRP3 inhibits AML progression by inducing PERK/eIF2-mediated apoptosis

**DOI:** 10.1186/s12964-024-01777-6

**Published:** 2024-09-02

**Authors:** Michela Luciano, Helene Sieberer, Peter W. Krenn, Hieu-Hoa Dang, Julia Vetter, Theresa Neuper, Diana Amend, Constantin Blöchl, Christian X. Weichenberger, Anna Eglseer, Michael S. Unger, Ancuela Andosch, Philip Steiner, Daniel Neureiter, Renate Bauer, Laura Hummer, Suzana Tesanovic, Stephanie Binder, Dominik P. Elmer, Helen Strandt, Susanne Schaller, Dirk Strunk, Lisa Pleyer, Richard Greil, Stephan Winkler, Tanja N. Hartmann, Dirk Schmidt-Arras, Christian G. Huber, Fritz Aberger, Jutta Horejs-Hoeck

**Affiliations:** 1 Department of Biosciences and Medical Biology, Paris-Lodron University Salzburg, Hellbrunner Strasse 34, Salzburg, 5020 Austria; 2 Cancer Cluster Salzburg, Salzburg, 5020 Austria; 3 Center for Tumor Biology and Immunology, Paris-Lodron University Salzburg, Salzburg, 5020 Austria; 4 Bioinformatics Research Group, University of Applied Sciences Upper Austria, Hagenberg Campus, Hagenberg, 4232 Austria; 5 Institute for Biomedicine, Eurac Research, Bolzano, 39100 Italy; 6 Institute of Pharmacology, Medical Faculty, Johannes Kepler University Linz, Linz, 4020 Austria; 7 Institute of Pathology, Paracelsus Medical University (PMU), University Hospital Salzburg (SALK), Salzburg, 5020 Austria; 8 Cell Therapy Institute, Spinal Cord Injury and Tissue Regeneration Center Salzburg (SCI-TReCS), Paracelsus Medical University (PMU), Salzburg, 5020 Austria; 9 Salzburg Cancer Research Institute (SCRI)-LIMCR, Salzburg, 5020 Austria; 10 3rd Medical Department with Hematology, Medical Oncology, Hemostaseology, Rheumatology and Infectiology, Oncologic Center, Paracelsus Medical University (PMU), University Hospital Salzburg (SALK), Salzburg, 5020 Austria; 11 Department of Medicine I, Medical Center, Faculty of Medicine, University of Freiburg, 79106 Freiburg, Germany

**Keywords:** Acute myeloid leukemia, Apoptosis, eIF2α, NLRP3, PERK

## Abstract

**Background:**

Acute myeloid leukemia (AML) is characterized by the abnormal proliferation of myeloid precursor cells and presents significant challenges in treatment due to its heterogeneity. Recently, the NLRP3 inflammasome has emerged as a potential contributor to AML pathogenesis, although its precise mechanisms remain poorly understood.

**Methods:**

Public genome datasets were utilized to evaluate the expression of NLRP3 inflammasome-related genes (IL-1β, IL-18, ASC, and NLRP3) in AML patients compared to healthy individuals. CRISPR/Cas9 technology was employed to generate NLRP3-deficient MOLM-13 AML cells, followed by comprehensive characterization using real-time PCR, western blotting, FACS analysis, and transmission electron and immunofluorescence microscopy. Proteomic analyses were conducted to identify NLRP3-dependent alterations in protein levels, with a focus on the eIF2 kinase PERK-mediated signaling pathways. Additionally, in vivo studies were performed using a leukemic mouse model to elucidate the pathogenic role of NLRP3 in AML.

**Results:**

Elevated expression of NLRP3 was significantly associated with diminished overall survival in AML patients. Genetic deletion, pharmacological inhibition and silencing by RNA interference of NLRP3 led to decreased AML cell survival through the induction of apoptosis. Proteomic analyses uncovered NLRP3-dependent alterations in protein translation, characterized by enhanced eIF2α phosphorylation in NLRP3-deficient AML cells. Moreover, inhibition of PERK-mediated eIF2α phosphorylation reduced apoptosis by downregulating pro-apoptotic Bcl-2 family members. In vivo studies demonstrated reduced leukemic burden in mice engrafted with NLRP3 knockout AML cells, as evidenced by alleviated leukemic symptoms.

**Conclusion:**

Our findings elucidate the involvement of the NLRP3/PERK/eIF2 axis as a novel driver of AML cell survival. Targeting NLRP3-induced signaling pathways, particularly through the PERK/eIF2 axis, presents a promising therapeutic strategy for AML intervention. These insights into the role of the NLRP3 inflammasome offer potential avenues for improving the prognosis and treatment outcomes of AML patients.

**Supplementary Information:**

The online version contains supplementary material available at 10.1186/s12964-024-01777-6.

## Background

Acute myeloid leukemia (AML) is a highly heterogenous hematopoietic disease induced by the oncogenic transformation of progenitor cells of the myeloid lineage, resulting in impaired hematopoiesis and dysregulated immune cell development [1]. Given that “tumor-promoting inflammation” is one of the hallmarks of cancer [2], it was suggested that deregulated inflammation may also play a key role in several aspects of AML, including disease progression and chemotherapy resistance [3].

Recent scientific advances highlighted the NLRP3 (NLR family pyrin domain containing 3) inflammasome as a promoter of chronic inflammation in myeloid cells [4]. The NLRP3 inflammasome is a key player of the innate immune system [5]. It is composed of NLRP3, the adapter protein ASC (Apoptosis-associated Speck-like protein containing CARD), and procaspase-1 [6]. Upon inflammasome formation, procaspase-1 undergoes proteolytic cleavage, and the resulting active caspase-1 cleaves the cytokine precursors pro-interleukin (IL)-1β and pro-IL-18 as well as the pore-forming gasdermin D into their mature forms, which in turn induce inflammation and pyroptotic cell death [7]. IL-1β has been reported to promote the growth of myeloid progenitor cells, contributing to an inflammatory environment and AML progression in a murine model [8]. Furthermore, oncogenic *KRAS* mutations were shown to induce NLRP3 via upstream activation of RAC1 GTPase and subsequent generation of reactive oxygen species (ROS), implicating upregulation of the RAC1/ROS/NLRP3/IL-1β axis as a crucial event in myeloproliferative disorders [9]. Accordingly, inhibition of NLRP3 inflammasome activation impaired AML cell proliferation in vitro, whereas upregulation of NLRP3 promoted AML progression and mortality in vivo in mice, identifying NLRP3 as a potential driver of AML [10].

Another hallmark of cancer is “sustained proliferative signaling” [2], which imposes a high demand on protein synthesis [11]. Global protein synthesis is initiated by the eukaryotic initiation factor 2 (eIF2) [12], which is composed of three subunits: α, β, and γ [13]. Phosphorylation of the α-subunit at serine 52 (p-eIF2α) during specific stress conditions by one of the four eIF2 kinases (PERK, PKR, GCN2, and HRI) [14] reduces global protein synthesis [12, 13]. Preventing eIF2α phosphorylation by loss-of-function mutations in these eIF2 kinases has been shown to promote malignant transformation in fibroblasts [15]. The eIF2 kinase PERK is activated by endoplasmic reticulum (ER) stress, resulting in eIF2 phosphorylation [16] and ATF4 activation. ATF4 in turn induces either apoptosis [17–19] by promoting expression of CHOP [20] and regulation of Bcl-2 family members [21] and/or autophagy by promoting the expression and conversion of LC3BI/II [22, 23]. The role of autophagy in tumorigenesis and AML remains controversial, with some studies indicating that high levels of autophagy promote AML disease development [24–27], while Watson et al. claim that autophagy limits leukemic progression [28]. In addition, it has been reported that autophagy inhibits NLRP3 inflammasome activation by eliminating inflammasome components [29].

Here we show that genetic deletion of NLRP3 in AML cells leads to PERK-mediated phosphorylation of eIF2α, which in turn activates intrinsic apoptotic pathways through upregulation of pro-apoptotic Bcl-2 family members in leukemic blast cells. This activation promotes both apoptosis and autophagy, ultimately leading to reduced AML cell survival in vitro and impaired disease progression in mice. In summary, our findings suggest that NLRP3 possesses anti-apoptotic functions by blocking PERK-mediated eIF2α phosphorylation, thereby promoting the survival of leukemic blasts in AML.

## Methods

Extensive details on the methods used can be found in the supplementary file.

### Primary human AML samples, cell lines and culture conditions

This study was conducted in accordance with established guidelines of the World Medical Association’s Declaration of Helsinki. AML cell lines and the human cervix carcinoma cell line HeLa were purchased from Leibniz-Institut DSMZ GmbH and cultured under standard conditions. All patient and healthy donor samples were obtained after approval from the Ethics Committee Salzburg (approval: 415-E/2009/2-2016 and approval: 415-E/1776/4-2014). NLRP3-deficient MOLM-13 (ΔNLRP3) cells were generated by CRISPR/Cas9 technology.

### Immunohistochemical staining and detection of cytokines

Immunohistochemistry was performed on prepared blocks of MOLM-13 wild-type (wt) and MOLM-13 ΔNLRP3 cells as well as on formalin-fixed paraffin-embedded (FFPE) BM trephine samples of normal controls and AML cases. Analysis of cytokine secretion in primary human AML samples and sera obtained from engrafted mice was performed using the Cytokine/Chemokine/Growth Factor 45-Plex Human ProcartaPlex™ system (ThermoFisher). Detection of intracellular protein expression was performed by western blotting.

### Cell proliferation, cell cycle and apoptosis analyses

Cell proliferation was assessed by using the eBioscience™ Cell Proliferation Dye eFluor™ 450 and cell numbers were determined using a Neubauer counting chamber. Cell cycle and apoptosis analyses were performed using FxCycle™ PI/RNase Staining Solution (Invitrogen, Catalog number: F10797) and Annexin V Apoptosis Detection Kits (Invitrogen, eBioscience by Thermo Fisher Scientific, Catalog number: 88-8006-74) according to the manufacturers’ instructions.

### qRT-PCR

For gene expression analysis the following primers were used: *NLRP3* primer pair (Sigma-Aldrich): forward 5’ -TCAGCACTAATCAGAATCTCACGCACCTTT − 3’ and reverse 5’ -CCAGGTCATTGTTGCCCAGGCTC − 3’; *CHOP* primer pair (Sigma-Aldrich): forward 5’ -CAAGAGGTCCTGTCTTCAGATGA − 3’ and reverse 5’-TCTGTTTCCGTTTCCTGGTCC-3’; *RPLP0* (house keeping/reference) primer pair: forward 5’ -GGCACCATTGAAATCCTGAGTGATGTG − 3’ and reverse 5’ -TTGCGGACACCCTCCAGGAAG − 3’.

### Animal studies

For the MOLM-13 tumor engraftment studies, NSG-S mice (MGI:6392814; male; age > 10 weeks) were intravenously injected with MOLM-13 CRISPR/Cas9 control (ctrl) cells or ∆NLRP3 cells and constantly monitored and scored for leukemia-associated changes in physical appearance, breathing rate and behavior. For the endpoint study, all mice of the same experiment were euthanized on the same day when predefined termination criteria were met. Peripheral blood, spleen and BM were isolated and evaluated for the percentage of human CD45^+^ cells by flow cytometry (CD45-PerCP-Cy5.5, Biolegend Catalog number: 304028; BD FACS Canto II). The mouse experiments were approved by the Government of Austria (BMWFW-66.012/0032-WF/V/3b/2017 and 2023 − 0.588.057).

### Proteomics

Cellular proteomes of MOLM-13 ctrl and ΔNLRP3 cells were prepared utilizing S-Trap columns (Protifi, USA) with small amendments to the manufacturer´s instructions. Acquired data were evaluated using MaxQuant (v1.6.3.4) to provide Uniprot entries and identified protein groups were imported into the R statistical programming language (v4.3.0). Graphs were generated with ggplot2 (v3.4.2), ShinyGO, and GraphPad Prism 8 software (GraphPad Software, San Diego, CA, USA).

### Transmission electron microscopy

MOLM-13 wt cells and MOLM-13 ∆NLRP3 cells were high-pressure frozen (HPF), cryosubstituted and subsequently embedded in epoxy resin. The embedded samples were trimmed and cut to ultrathin sections (~ 70 nm). Sections on Formvar-coated copper grids were then transferred to the transmission electron microscope (TEM) and images were recorded on a LEO 912 AB Omega TEM at 80 kV.

### Database analysis

Public genome datasets GSE13159 and GSE12417 from NCBI’s Gene Expression Omnibus (NCBI-GEO) were used to evaluate expression of *IL1B*,* IL18*,* ASC*, and *NLRP3* in AML patients and in healthy individuals. Patients in the GSE12417 dataset were treated according to the outdated AMLCG-1999 protocol, which included 6-thioguanine/cytarabine/daunorubicin and/or high-dose cytarabine/mitoxantrone regimens [30].

### Immunofluorescence and confocal microscopy

Cytospin samples were generated by using a Cytospin 4 centrifuge (Epredia). Cells were analyzed using a Zeiss Observer Z1 fluorescence microscope equipped with an Abberior Instruments STEDYCON unit for confocal and super-resolution STED microscopy. Representative confocal z-stacks were taken with a 100× objective. Images show maximum intensity projections and were post-processed with Fiji (ImageJ1.54f) and Microsoft PowerPoint.

### Statistical analysis

Mice were randomly assigned to the experimental groups. No blinding was used for the injection or analysis. There were no exclusion criteria for animals. Statistical analyses were performed with GraphPad Prism 8 software (GraphPad Software, San Diego, CA, USA). Data was tested for normality and appropriate statistical tests were used: Statistical analyses were performed by Wilcoxon matched-pairs signed rank test, a paired or two-tailed unpaired t-test for the analysis between two groups, one-way ANOVA with Tukey’s post-hoc test for multiple comparisons, a two-way ANOVA with Tukey’s or Šídák’s post-hoc test was performed for multiple comparisons and a Cox proportional hazards model was used for the statistical analysis of the Kaplan-Meier curve. Significance levels are defined as follows: *, *p* ≤ 0.05; **, *p* ≤ 0.01; ***, *p* ≤ 0.001; ****, *p* ≤ 0.0001; ns, not significant.

## Results

### Increased levels of NLRP3 inflammasome components and downstream effectors in AML patients

**Fig. 1:**
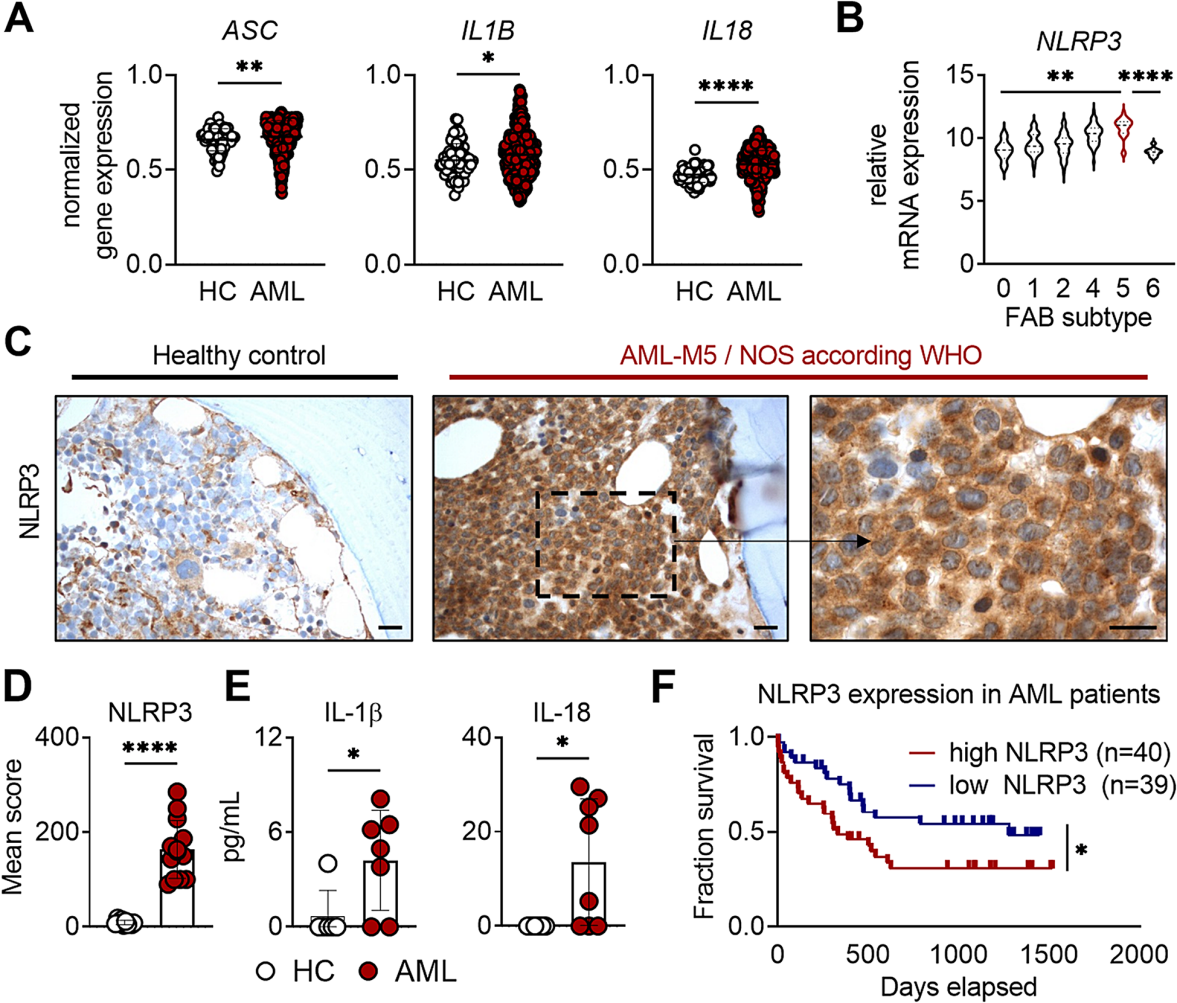
Increased expression of NLRP3-related effectors in AML patients. **A** *ASC*,* IL1B and IL18* normalized gene expression in acute myeloid leukemia (AML) patients (AML, *n* = 542) and healthy controls (HC, *n* = 74) in the bone marrow were determined using the publicly available dataset GSE13159. **B*** NLRP3* expression analysis within the AML subgroups using the public genome dataset GSE12417 (AML FAB 0, *n* = 6; AML FAB 1, *n* = 68; AML FAB 2, *n* = 79; AML FAB 4, *n* = 53; AML FAB 5, *n* = 25; AML FAB 6, *n* = 9). The horizontal line defines the median. **C**,** D** Representative immunohistochemistry staining for NLRP3 in FFPE bone marrow trephine biopsies (**C**) and mean NLRP3 expression score (**D**) are shown of HC (*n* = 10) and AML cases (*n* = 13; M5 = FAB classification). Scale bars correspond to 20 μm. **E** IL-1β and IL-18 secretion in supernatants of bone marrow mononuclear cells (BM-MNCs) isolated from AML patient samples (AML, *n* = 8) and HC (*n* = 6) were detected by multiplex bead-based immunoassay after 24 h of in vitro culture. **F** Kaplan-Meier curve analysis of the dataset GSE12417 (platform GPL570) comparing the survival of AML patients having high NLRP3 expression (*n* = 40) vs. low NLRP3 expression (*n* = 39). Data were tested for normality and appropriate statistical tests were used: a Mann-Whitney U test (**A**, **D**) or a two-tailed, unpaired t-test (**C**) was used for comparing two groups, a one-way ANOVA with Tukey’s post-hoc test was used for multiple comparisons (**B**), while a Cox proportional hazards model was used for the Kaplan-Meier curve (**E**). Dots represent individual donors and error bars represent mean ± SD. Significance levels are defined as follows: *, *p* ≤ 0.05; **, *p* ≤ 0.01; ***, *p* ≤ 0.001; ****, *p* ≤ 0.0001

A recent study by Hamarsheh et al. showed that oncogenic *KRAS*-mediated myeloproliferation is dependent on NLRP3 expression and activation in mice [9], suggesting a potential role of NLRP3 in leukemogenesis. Similarly, Zhong et al. reported that NLRP3 and the inflammasome components ASC, caspase-1 and the downstream effector IL-1β were overexpressed and hyperactivated in bone marrow mononuclear cells (BM-MNC) in a cohort of AML patients [10]. We extended these observations by using a publicly available gene array dataset (NCBI-GEO dataset GSE13159) containing 542 AML patient samples. Our bioinformatic analysis confirmed significantly higher gene expression of the inflammasome component *ASC* along with the downstream effectors *IL1B* and *IL18* in the bone marrow of AML patients compared to healthy control (HC) samples (Fig. 1A). Interestingly, the expression of inflammasome-related members of the NLR family remained unaltered in this AML patient cohort comprising a mixture of FAB subtypes (Suppl. Figure 1). A more detailed analysis revealed that NLRP3 mRNA expression was significantly increased in patient samples categorized as monocytic M4 and M5 FAB subtypes compared to undifferentiated (M0) or erythroblastic leukemia (M6) (Fig. 1B, data from the NCBI-GEO GSE12417, platform GPL570). In line with these observations, primary bone marrow (BM) samples from M5 AML patients showed significantly elevated NLRP3 protein levels compared to healthy controls (Fig. 1C/D) and bone marrow mononuclear cells (BM-MNCs) from AML patients secreted significantly higher amounts of IL-1β and IL-18 (Fig. 1E). Analysis of AML patient survival revealed a significantly higher median overall survival among patients with low NLRP3 expression (*n* = 39) compared to those with high (*n* = 40) NLRP3 expression (NCBI-GEO GSE12417) (Fig. 1F), supporting the hypothesis that NLRP3 plays a critical role in AML pathogenesis.

### Targeting NLRP3 induces apoptosis in AML cells

**Fig. 2:**
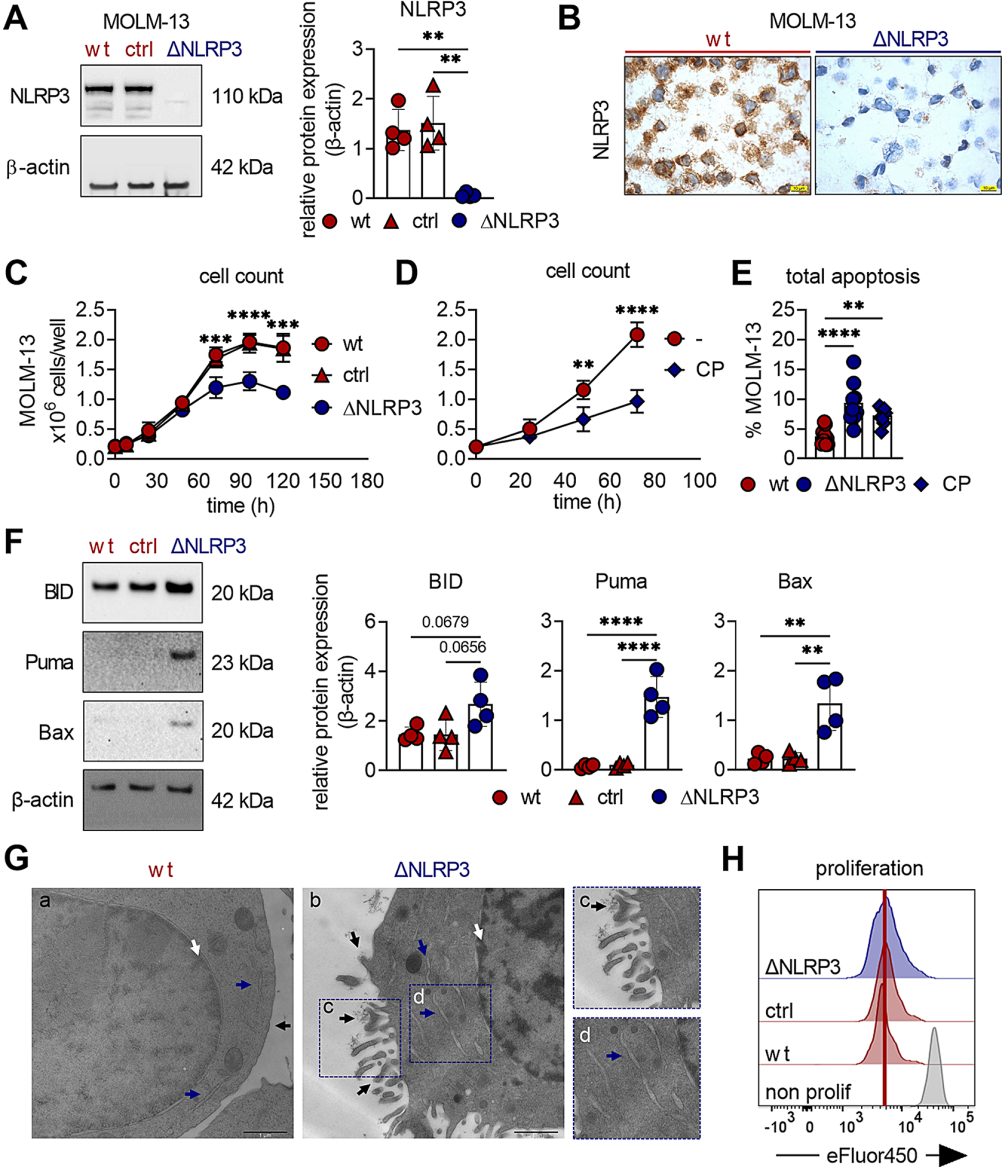
Targeting NLRP3 induces apoptosis in AML cells. **A** NLRP3 protein levels were determined in MOLM-13 wild-type (wt), CRISPR/Cas9 control off-NLRP3-target (ctrl) and CRISPR/Cas9 NLRP3 knockout (ΔNLRP3) cells by Western blot analysis including β-actin as a loading control (*n* = 3). **B** Immunohistochemical staining of NLRP3 was performed in MOLM-13 wt and ΔNLRP3 cells (scale bars correspond to 20 μm). **C** Cell counts of MOLM-13 wt, ctrl, and ΔNLRP3 were determined at the indicated time points of culture (*n* = 6). Asterisks indicate significant differences between wt and ΔNLRP3 cells. Mean ± SD is shown. **D** Cell counts of untreated MOLM-13 wt (-) and wt cells treated with 125 µg/mL of the NLRP3 inhibitor CP-456,773 (CP) were determined at the indicated incubation time points (*n* = 3). Mean ± SD is shown. **E** Bar chart showing the percentage of apoptotic MOLM-13 wt, ΔNLRP3 cells and wt cells treated with 125 µg/mL CP-456,773 (CP) 48 h after seeding (*n* = 9). **F** Western blot showing the expression of pro-apoptotic proteins (BID, Puma, and Bax) and β-actin (loading control) in MOLM-13 wt, ctrl, and ΔNLRP3 cells 48 h after seeding (*n* = 4) and densiometric quantification relative to the loading control β-actin. **G** Transmission electron micrographs of MOLM-13 wt (panel a) and ∆NLRP3 (panel b) 48 h after seeding. Scale bars correspond to 1 μm. In panel b, the black arrows indicate cellular shrinkage, membrane blebbing, and apoptotic bodies; blue arrows highlight the presence of swollen endoplasmic reticulum (ER), and the white arrow indicates the occurrence of pyknosis. Zoomed regions of interest of ΔNLRP3 cells are shown on the very right-hand side (panel c and d). **H** Proliferation of MOLM-13 wt, ctrl, and ΔNLRP3 cells (*n* = 7) was monitored 48 h after seeding by flow cytometry. One representative histogram is shown. The vertical red line indicates the fluorescence peak of proliferating MOLM-13 wt cells. Freshly stained MOLM-13 wt cells served as a negative control (grey peak). Dots in graphs indicate individual replicates; bars represent mean ± SD. One-way ANOVA with Tukey’s post-hoc test was performed for multiple comparisons (**A, E, F**), two-way ANOVA with Tukey’s (**C**) or Šídák’s post-hoc test (**D**) was performed for multiple comparisons. Significance levels are defined as follows: *, *p* ≤ 0.05; **, *p* ≤ 0.01; ***, *p* ≤ 0.001; ****, *p* ≤ 0.0001; ns, not significant

To investigate the potential myeloproliferative role of NLRP3 in more detail, we made use of the highly aggressive AML cell line MOLM-13, which exhibits high endogenous NLRP3 expression, and generated NLRP3-deficient (ΔNLRP3) and non-targeting control (ctrl) MOLM-13 cells by CRISPR/Cas9 technology. MOLM-13 ΔNLRP3 cells showed significantly reduced NLRP3 mRNA (Suppl. Figure 2A) and protein levels (Fig. 2A + B), confirming successful knockout of NLRP3. Strikingly, after a culture period of 2–5 days, we observed significantly lower cell counts for ΔNLRP3 cells compared to MOLM-13 wild-type (wt) cells, while the ctrl cell counts remained comparable to those of the wt cells throughout the observation period (Fig. 2C). Similar to ΔNLRP3 cells, treatment with the NLRP3 inflammasome inhibitor CP-456,773 markedly diminished the cell counts of MOLM-13 wt cells (Fig. 2D) as well as the secretion of IL-β upon inflammasome activation (Suppl. Figure 2B). We additionally tested CP-456,773 in MOLM-13 and MV4-11 (AML cell lines), HeLa (cervical cancer cell line) and human primary monocytes characterized by varying NLRP3 and ASC expression levels (Suppl. Figure 2C). Cell numbers of the two AML cell lines decreased with increasing concentrations of the inhibitor (Suppl. Figure 2D). Conversely, HeLa cells, which do not express NLRP3, and primary human monocytes with basal NLRP3 expression remained unaffected (Suppl. Figure 2D).

Next, we investigated if reduced survival contributes to the reduced cell numbers in MOLM-13 ΔNLRP3 cells. As shown in Fig. 2E, both ∆NLRP3 and NLRP3 inhibitor-treated wt cells exhibited considerably higher levels of apoptotic cell death compared to wt cells. Similarly, NLRP3 silencing by RNA interference also resulted in higher apoptosis in OCI-AML3 cells (Suppl. Figure 2E). Furthermore, we detected increased levels of the pro-apoptotic proteins BID, Puma and Bax in MOLM-13 ΔNLRP3 cells (Fig. 2F). Transmission electron microscopy (TEM) revealed that ΔNLRP3 cells exhibited morphological hallmarks of apoptosis, including pyknosis, swollen endoplasmic reticula (ER), and apoptosis-related membrane blebbing (Fig. 2G, panel b, c and d), which were not present in wt cells (Fig. 2G, panel a). Surprisingly, proliferation (Fig. 2H, Suppl. Figure 2F) and also cell-cycle profiles (Suppl. Figure 2G) of MOLM-13 ∆NLRP3 cells were unaffected when compared to wt cells. These findings suggest that the reduced cell counts (Fig. 2C) are mainly caused by increased apoptosis and not due to alterations in cell proliferation. Taken together, these experiments show that both genetic deletion and pharmacologic inhibition of NLRP3 induce apoptosis in AML cells. This indicates that elevated NLRP3 expression as observed in leukemic cells of AML patients (Fig. 1C/D), prevents apoptosis and promotes AML cell survival.

### NLRP3 deletion induces enrichment of proteins associated with the translation pathway

**Fig. 3:**
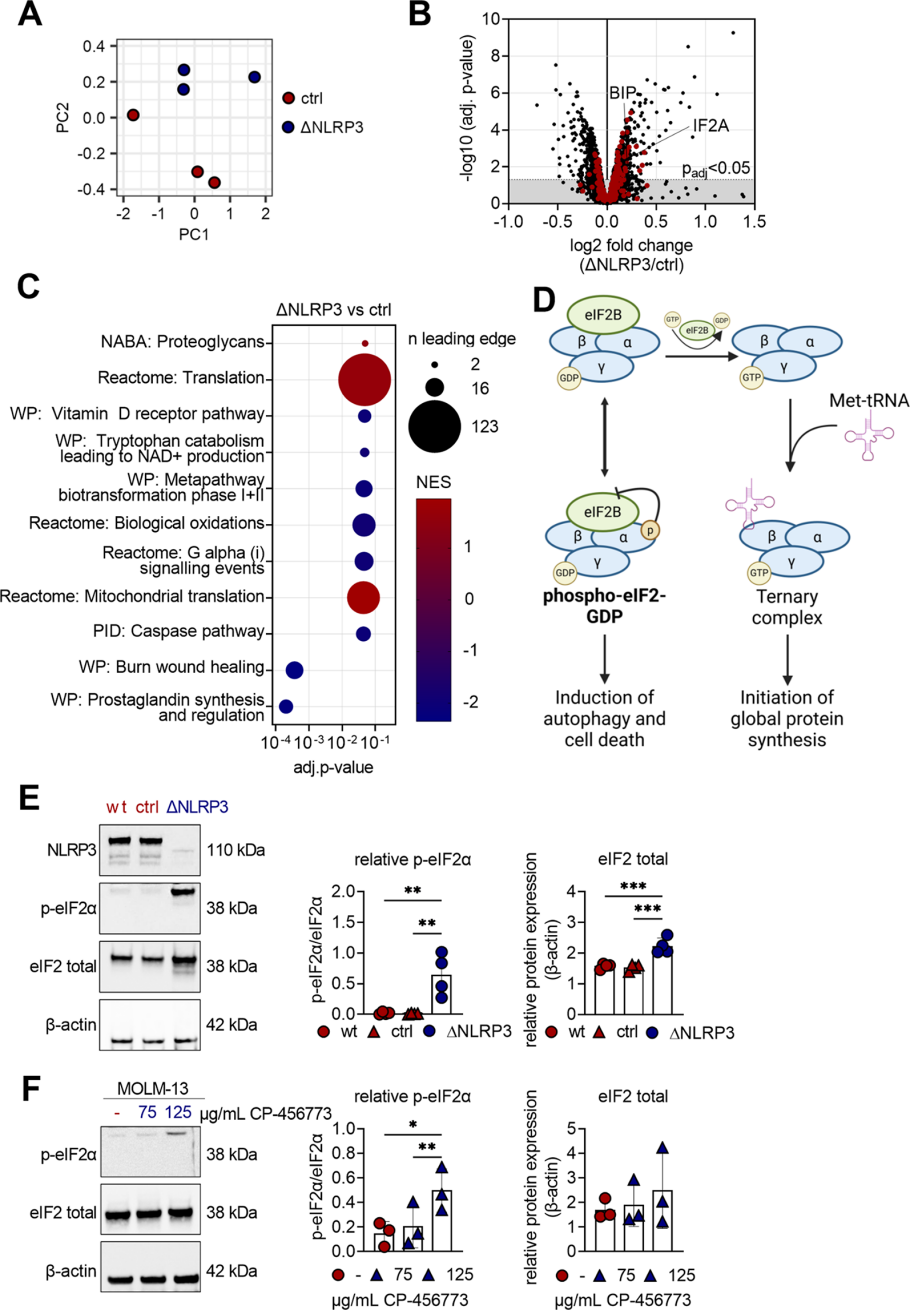
Deletion of NLRP3 modulates translation in AML cells. **A** Principal component analysis of proteome data of MOLM-13 CRISPR/Cas9 control (ctrl) and CRISPR/Cas9 NLRP3 knockout (ΔNLRP3) cells (cultured for 24 h) after HPLC-MS analysis (*n* = 3). **B** Volcano plot of cleaned proteins. The dashed horizontal line indicates the adjusted p-value cut-off < 0.05 (*n* = 3859; red = proteins assigned to the Reactome “Translation” pathway). **C** GSE analysis revealing significantly differentially regulated pathways between MOLM-13 ctrl and ΔNLRP3 cells. **D** Graphic illustration of the EIF2 pathway, including involved proteins and global downstream effects. Illustration was created with BioRender.com. **E** MOLM-13 wild-type (wt), ctrl and ΔNLRP3 were cultured for 24 h before being processed for Western blot analysis of NLRP3, p-eIF2α, eIF2 total and β-actin (*n* = 4). **F** MOLM-13 wt cells were either left untreated (-) or treated with 75–125 µg/mL CP-456,773 for 24 h before being processed for Western blot analysis of NLRP3, p-eIF2α, eIF2 total and β-actin (*n* = 3). For E + F, the ratio of phosphorylated to total eIF2 was calculated. β-actin was used as a loading control and for the densiometric quantification of eIF2 total. One-way ANOVA with Tukey’s post-hoc test (**E + F**) was performed for multiple comparisons. Significance levels are defined as follows: *, *p* ≤ 0.05; **, *p* ≤ 0.01; ***, *p* ≤ 0.001

To further understand how loss of NLRP3 induces apoptosis, we performed differential proteomics analyses by using high-performance liquid chromatography (HPLC) employing micro pillar array columns (µPAC) in combination with Orbitrap mass spectrometry (MS) on MOLM-13 ctrl and ΔNLRP3 cells. The proteomics dataset output by MaxQuant contained 4286 proteins, and after dataset cleaning, we arrived at a purged dataset with 3859 unique proteins. Principal component analysis (PCA) shows that the measurements obtained from ΔNLRP3 are separated from the control by principal component 2, with a certain degree of variability between the biological replicates (Fig. 3A), but no apparent batch effects. We chose cyclic loess normalization, which performed marginally better than the other methods offered by the NormalyzerDE web interface, and then subjected the data to differential abundance analysis with NormalyzerDE, giving evidence for 171 downregulated and 161 upregulated proteins (Fig. 3B). Gene set enrichment analysis for 3090 canonical pathways identified 11 enriched pathways (Fig. 3C). The Reactome “Translation” pathway included the highest number of differentially regulated proteins between ΔNLRP3 and ctrl cells and is significantly upregulated in ΔNLRP3 cells (Fig. 3C). The fact that increased protein synthesis is a pre-requisite for ER stress-mediated apoptosis [31] is in line with our findings that targeting NLRP3 is associated with enrichment of the translation pathway (Fig. 3C) as well as markers for ER stress (swollen ERs (Fig. 2G) and elevated levels of ER-related chaperone BiP (Fig. 3B)) and apoptosis (Fig. 2E). Further analysis of the top 100 significantly up- and downregulated proteins revealed that the EIF2 signaling complex, known to be involved in protein translation, was one of the most affected pathways (Suppl. Figure 3A). As illustrated in Fig. 3D, phosphorylation of eIF2α, an important protein of the EIF2 signaling complex, is a pivotal regulator for the initiation of translation, apoptosis, and autophagy. To investigate whether NLRP3-dependent translational alterations were mediated via eIF2α, we analyzed eIF2α phosphorylation at serine 51 in MOLM-13 wt, ctrl and ΔNLRP3 cells. Strikingly, we observed enhanced phosphorylation (Fig. 3E + F) and increased total eIF2α levels (Fig. 3B + E) in ΔNLRP3 cells compared to wt and ctrl cells. Additionally, treatment of MOLM-13, MV4-11, OCI-AML3 and THP-1 cells (AML patient-derived cell lines) with the NLRP3 inhibitor CP-456,773 induced significantly higher phosphorylation of eIF2α compared to their untreated counterparts (Fig. 3F, Suppl. Figure 3C–E). Taken together, this indicates that NLRP3 dampens eIF2α phosphorylation in AML cells, which may in turn modulate translation processes.

### The PERK/eIF2 axis drives apoptosis and autophagy in ΔNLRP3 cells

**Fig. 4:**
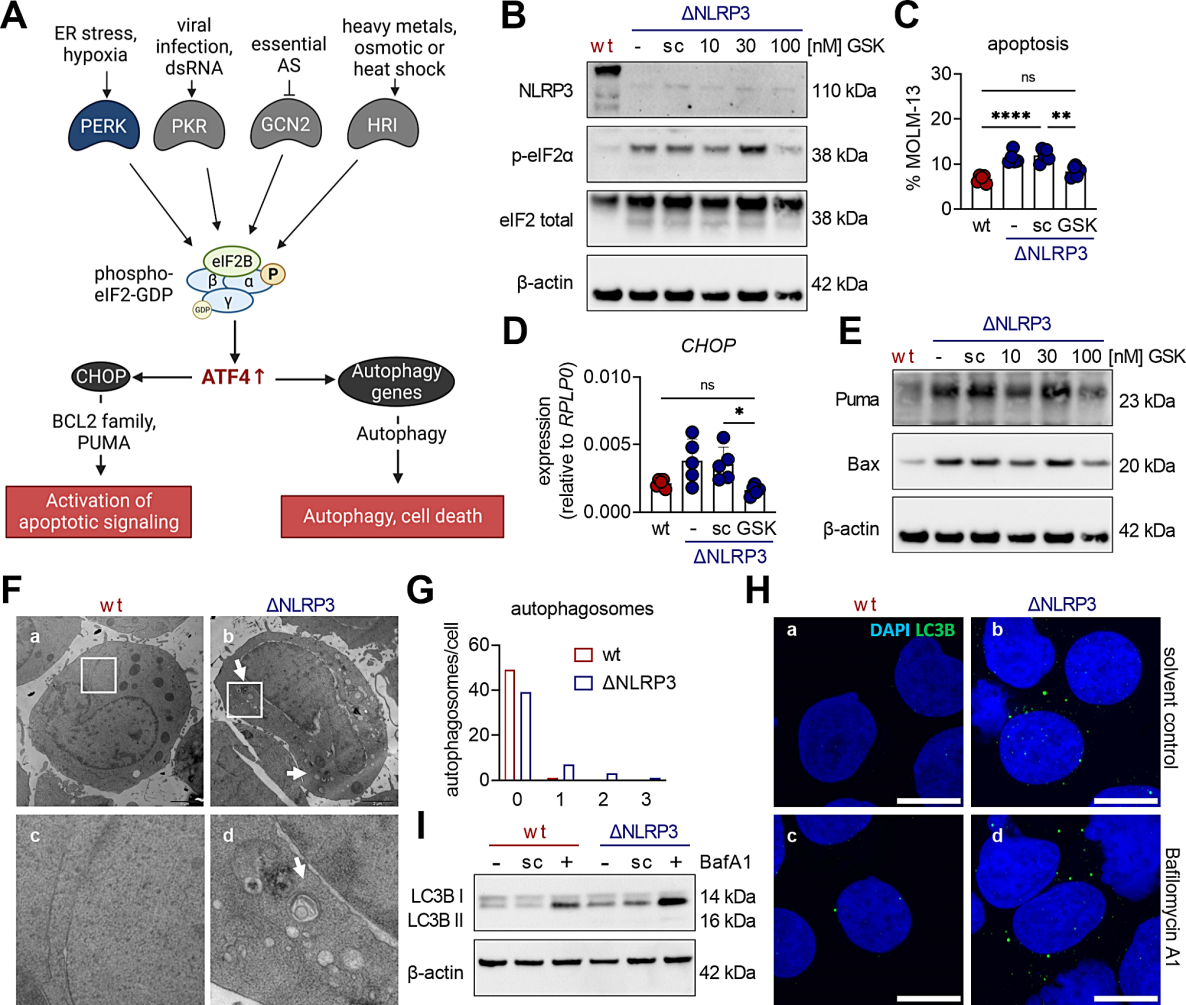
The PERK/eIF2 axis promotes apoptosis and autophagy in ΔNLRP3 cells. **A** Graphic illustration of eIF2 signaling and downstream effects. eIF2 kinases phosphorylate eIF2α at serine 51 during specific stress conditions and thereby activate downstream effectors such as ATF4 and CHOP, ultimately resulting in autophagy and/or cell death. Illustration was created with BioRender.com. **B** Untreated MOLM-13 wt, or ΔNLRP3 cells subjected to either no treatment (-), 0.1% DMSO (solvent control = sc), or increasing concentrations of the PERK inhibitor GSK2606414 (GSK) for 48 h were analyzed by Western blot for protein expression of NLRP3, phospho-eIF2α (Ser51), eIF2 total and β-actin (loading control) (*n* = 4). **C** Untreated MOLM-13 wt, and ΔNLRP3 cells were subjected to either no treatment (-), 0.1% DMSO (solvent control = sc), or 100 nM GSK2606414 (GSK) and incubated for 48 h before apoptotic rates were determined using flow cytometry (*n* = 5). **D** MOLM-13 cells were cultured as described in (**C**) before being processed for Western blot analysis of Puma, Bax, and β-actin (loading control) (*n* = 3). **E** *CHOP* mRNA levels were determined by qRT-PCR (*n* = 5, relative mRNA expression to the housekeeping gene *RPLP0*) in MOLM-13 cells that were cultured as described in (**C**). **F** Transmission electron micrographs of MOLM-13 wt (panel a and c) and ∆NLRP3 (panel b and d) cultured for 48 h. Arrows indicate the presence of autophagosomes in ΔNLRP3 cells (panel b). Panel c and d represent enlargements of the framed regions of panels a and b. Scale bar: 2 μm. **G** TEM images of 50 MOLM-13 wt and 50 ΔNLRP3 cells were analyzed for the presence of autophagosomes. Bar graph showing the number of counted cells with either 0, 1, 2, or 3 autophagosomes after 48 h incubation. **H** MOLM-13 wt and ΔNLRP3 cells were incubated for 48 h and 3 h before harvest either treated with 0.1% DMSO (solvent control; panels a and b) or 100 nM Bafilomycin A1 (panels c and d). Immunofluorescence staining for LC3B (green), and confocal fluorescence microscopy were performed. DAPI (blue) was used to stain cell nuclei. Scale bar: 10 μm. **I** MOLM-13 wt and ΔNLRP3 cells were cultured as described in (**H**). The cells were then subjected to Western blot analysis of LC3B and β-actin (loading control) (*n* = 4). Dots indicate individual experiments; bars represent mean ± SD. For statistical analyses, one-way ANOVA with Tukey’s post-hoc test was performed for multiple comparisons (**C** + **D**). Significance levels are defined as follows: *, *p* ≤ 0.05; **, *p* ≤ 0.01; ****, *p* ≤ 0.0001; ns, not significant

Four kinases (PERK, PKR, GCN2, HRI) are known to phosphorylate eIF2α during specific stress conditions (Fig. 4A) [13]. During ER stress, activation of PERK in particular leads to eIF2α phosphorylation. The finding that ΔNLRP3 cells have swollen ERs (Fig. 2H) and show upregulation of the ER stress sensor BiP (Fig. 3B), both indicative of ER stress, prompted us to analyze the effects of PERK in our setting. PERK-induced eIF2α phosphorylation is known to cause increased ATF4 levels and subsequent expression of CHOP and pro-apoptotic Bcl-2 family members, which ultimately culminates in apoptosis and/or autophagy (Fig. 4A) [32].

Treatment of ΔNLRP3 cells with increasing concentrations of the PERK inhibitor GSK2606414, most notably at 100 nM, resulted in a decrease in eIF2α phosphorylation (Fig. 4B, Suppl. Figure 4A), indicating that PERK plays an important role in phosphorylating eIF2α in ΔNLRP3 cells. In addition to increased apoptosis rates (Figs. 2E and 4C) and elevated *CHOP* gene expression (Fig. 4D), we also observed increased levels of pro-apoptotic proteins (Figs. 2F and 4E) in ΔNLRP3 cells, however, all of these effects were reduced upon PERK inhibition (Fig. 4C-E, Suppl. Figure 4B). This set of experiments indicates that the PERK/eIF2 axis regulates apoptosis in ΔNLRP3 cells.

Furthermore, the PERK/eIF2 axis additionally induces autophagy by mediating LC3 conversion [22]. Therefore, we next investigated whether ΔNLRP3 cells also showed higher levels of autophagy compared to wt cells. TEM images confirmed this hypothesis as we found higher numbers of autophagosomes (Fig. 4F/G) and LC3B puncta in immunofluorescence images in ΔNLRP3 cells (Fig. 4H). Furthermore, ΔNLRP3 cells displayed increased lipidated LC3B-II in the presence of the autophagy inhibitor bafilomycin compared to MOLM-13 wt cells (Fig. 4I, Suppl. Figure 4 C). Therefore, we conclude that the increased autophagic flux in ΔNLRP3 cells is mediated by elevated eIF2α phosphorylation. Taken together, our data demonstrate that NLRP3 expression antagonizes PERK/eIF2-induced apoptosis and autophagy in AML cells, thereby promoting cell survival.

### Loss of NLRP3 attenuates leukemia development in vivo

**Fig. 5:**
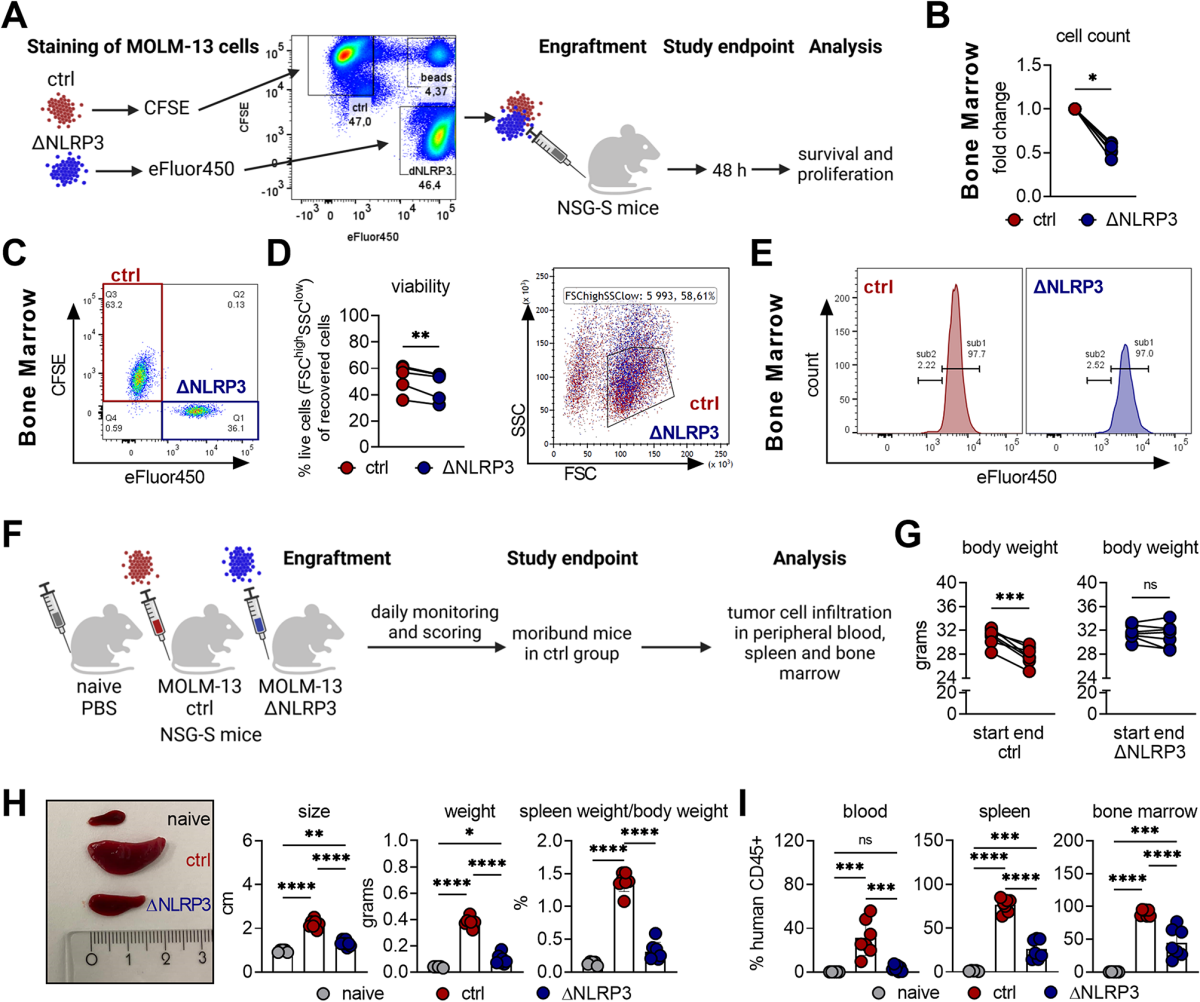
NLRP3 promotes AML development and progression in vivo. **A** Viable MOLM-13 ctrl and ΔNLRP3 cells were labeled with the cell tracker dyes CFSE or eFluor450, respectively or vice versa, mixed and injected into NSG-S mice. Quantification of human CD45^+^ dye^+^ cells was performed 48 h after engraftment to assess survival and proliferation. Illustration was created with BioRender.com. **B/C** Relative cell counts (**B**) and flow cytometric analysis (**C**) of CFSE- or eFluor450-labeled ΔNLRP3 or ctrl cells in the BM of NSG-S mice. For both ctrl and ΔNLRP3 samples, *n* = 6. **D** Cell viability, as determined by FSC^high^SSC^low^ gating strategy, of CFSE- or eFluor450- labeled ΔNLRP3 or ctrl cells. **E** Proliferation of ctrl cells and ΔNLRP3 cells in the BM of NSG-S mice determined by eFluor450 dye dilution. **F** NSG-S mice that received PBS (naïve), MOLM-13 ctrl, or ΔNLRP3 cells were monitored for leukemia development and analyzed for tumor cell infiltration. Illustration was created with BioRender.com. **G** Body weight of NSG-S mice was determined at the day of engraftment (start) and at the study endpoint (end). **H** The length and weight of the spleen and the organ-body-index were determined at the study endpoint. **I** Percentages of human CD45^+^ cells in peripheral blood (PB), spleen and BM measured by flow cytometry at the study endpoint. Naïve samples, *n* = 5; ctrl samples, *n* = 7; ΔNLRP3 samples, *n* = 7. Data were tested for normality and appropriate statistical tests were used: Statistical analyses were performed by Wilcoxon matched-pairs signed rank test (**B**), a paired t-test for the analysis between two groups (**D**, **G**) or one-way ANOVA with Tukey’s post-hoc test for multiple comparisons (**H, I**). Data in panels H and I are shown as mean ± SD from two independent experiments. Significance levels are defined as follows: *, *p* ≤ 0.05; **, *p* ≤ 0.01; ***, *p* ≤ 0.001; ****, *p* ≤ 0.0001; ns, not significant

To evaluate the impact of NLRP3 deletion on early organ infiltration, tissue-specific survival in a non-tumor-primed environment, and proliferation during leukemic cell seeding into bone marrow and spleen, we conducted a competitive experiment by injecting equal amounts of carboxyfluorescein succinimidyl ester (CFSE)-labeled MOLM-13 ctrl cells and eFluor 450 (eFluor450)-labeled ΔNLRP3 cells (or vice versa) into immunodeficient NSG-S mice. Human CD45-positive/dye-positive cells were quantified in the BM, peripheral blood (PB), and spleen 48 h post-injection (Fig. 5A). In line with reduced survival of NLRP3-deficient MOLM-13 cells in vitro (Fig. 2C), we observed significantly fewer ΔNLRP3 cells in the bone marrow (Fig. 5B/C), PB, and spleen (Suppl. Figure 5A) compared to MOLM-13 ctrl cells, thus suggesting impaired survival of ΔNLRP3 cells in vivo. Accordingly, the number of viable MOLM-13 ΔNLRP3 cells (FSC^high^SSC^low^) was significantly reduced compared to ctrl cells, not only in the BM (Fig. 5D) but also in the non-supportive spleen environment and PB (Suppl. Figure 5A). Consistent with our previous in vitro proliferation analysis (Fig. 2H), cell proliferation of MOLM-13 ΔNLRP3 cells was not altered compared to that of the ctrl cells in vivo (Fig. 5E and Suppl. Figure 5B). Therefore, we decided to perform a long-term engraftment experiment by transplanting MOLM-13 ctrl or MOLM-13 ΔNLRP3 cells into NSG-S mice, which were monitored for leukemia development. NSG-S mice injected with PBS only were used as a healthy, reference cohort (naïve). While the tumor onset in this aggressive in vivo leukemia model was delayed according to peripheral blood tumor load checks at day 11 after injection (Suppl. Figure 5 C), overall survival was only slightly increased in mice receiving ΔNLRP3 cells compared to mice receiving control cells (Suppl. Figure 5D). However, our endpoint study (Fig. 5F) revealed that mice engrafted with NLRP3-positive MOLM-13 ctrl cells exhibited a significant reduction in body weight and severe spleen enlargement, while mice engrafted with MOLM-13 ΔNLRP3 cells experienced neither weight loss nor severe splenomegaly (Fig. 5G + H) compared to the naïve control cohort. In addition, mice engrafted with MOLM-13 ΔNLRP3 cells showed reduced percentages of total human CD45^+^ cells in PB, spleen, and BM compared to mice engrafted with MOLM-13 ctrl cells (Fig. 5I). In accordance with the reduced survival of ΔNLRP3 leukemia cells in recipient mice (Fig. 5D), analysis of serum showed significantly lower secretion of human growth factors and cytokines such as TNFα, PIGF-1, HGF and the inflammasome-related cytokine IL-18, known to have a vital role in AML, when compared to control recipient mouse serum (Suppl. Figure 5E). Taken together, these findings show that NLRP3 is a critical driver of leukemia development and AML progression in vivo.

## Discussion

Important evidence for involvement of NLRP3 — a central hub of innate immunity that mediates the release of pro-inflammatory mediators — in human cancers emerged from a study showing that 15 out of 24 disparate, mostly solid, cancers had significantly different expression profiles of NLRP3 and other inflammasome-related genes compared to normal tissue [33]. It also became clear that NLRP3 could play dual roles in cancer, being either tumor suppressive, as observed in colitis-associated colorectal cancer, or tumor-promoting, especially evident in cancers of the skin, breast, and stomach [34, 35]. Furthermore, it was shown that the deregulation of the NLRP3 inflammasome can cause hematopoietic diseases [9, 10, 36–38]. A leukemogenic role for the NLRP3 inflammasome has only recently been proposed [9, 10]. Yet, the underlying mechanisms as to how NLRP3 contributes to the pathogenicity of AML are still not fully understood.

In the present study, we confirmed a potential myeloproliferative role of NLRP3 by demonstrating that the expression of *NLRP3* and the NLRP3 inflammasome-related genes *ASC*, *IL1B*, and *IL18* is increased in AML patient cells compared to cells from healthy donors. In concordance, Hamarsheh et al. identified oncogenic KRAS signaling as a driver of NLRP3 activation and reported particularly high levels of cleaved caspase-1 and increased IL-1β production in *KRAS*^*mut*^ peripheral blood mononuclear cells (PB-MNC) from AML patients compared to non-*KRAS* mutated PB-MNCs from AML patients [9]. Our study extends this observation by showing enhanced NLRP3 expression in BM-MNCs of AML patients, which also aligns with recent findings of Zhong et al. [10]. We additionally showed that high NLRP3 expression correlates with poor overall survival in AML patients, again highlighting a prognostic role of NLRP3 in AML.

To further study the functional role of NLRP3 in AML, we used CRISPR/Cas9 technology to generate an NLRP3-deficient human AML (MOLM-13) cell line, which was characterized by reduced cell survival in vitro. Previous studies suggested that NLRP3 inflammasome activation induces cell proliferation in different disease models, including AML [10, 39–42] but we observed no effects on cell proliferation when NLRP3 was deleted. In contrast, we found that genetic deletion of NLRP3 induced apoptosis by upregulation of pro-apoptotic Bcl-2 family members, suggesting that the NLRP3 protein may promote cell survival, but not cell proliferation. Consistently, pharmacological inhibition as well as silencing of NLRP3 corroborated our data obtained in MOLM-13 ΔNLRP3 cells, as we observed increased apoptosis in AML cell lines in which NLRP3 is inhibited or silenced, respectively.

To identify potential mechanisms for the induction of apoptosis in ΔNLRP3 cells, we performed shotgun proteome analysis, which revealed enrichment of the “Translation” pathway accompanied by enhanced phosphorylation of eIF2α in MOLM-13 ΔNLRP3 cells. We have further confirmed this novel link between NLRP3 and eIF2 signaling in additional AML cell lines, including OCI-AML3, MV4-11, THP-1 and MOLM-13, as we show that inhibition of NLRP3 leads to increased eIF2α phosphorylation. This is critical, because not only is phospho-eIF2α a pivotal regulator of protein synthesis [43], but stress-induced phosphorylation of eIF2α induces cell death [44], which is in line with our finding as enhanced phosphorylation of eIF2α correlated with increased apoptosis in ΔNLRP3 cells. In addition, Han et al. showed that increased translation is a prerequisite of ER stress-mediated apoptosis induced by ATF4 and CHOP [31]. Our observations agree with these findings, as we detected enrichment of proteins of the “Translation” pathway and elevated levels of *CHOP* and apoptosis in ΔNLRP3 cells. Furthermore, we postulate that ΔNLRP3 cells experience increased ER stress leading to activation of the PERK/eIF2 axis, as evidenced by elevated levels of the ER stress sensor BiP and by the presence of swollen ER. This hypothesis is supported by studies demonstrating that ER stress is associated with ER swelling [45–47], accumulation of BiP [48], and activation of the eIF2 kinase PERK [16]. Of note, we showed here that PERK mediates eIF2α phosphorylation and subsequent induction of apoptosis, because the increased phosphorylation of eIF2α, elevated levels of *CHOP*, pro-apoptotic proteins, and apoptosis that we observed were all reduced upon pharmacological inhibition of PERK in ΔNLRP3 cells. This is in line with the study from Han et al. reporting that ER stress-induced cell death is mediated by the transcription factors ATF4 and CHOP, which act as downstream effectors of phosphorylated eIF2α [31].

Our findings of a molecular signaling network in AML linking ER stress to apoptosis are further supported by recent in vitro studies reporting that certain drugs (e.g.: venetoclax combined with metformin, GSK-J4, and camalexin) induce apoptosis in human AML cells through induction of ER stress [49–51]. In a therapeutic context, we further provide evidence that targeting the phosphorylation of eIF2 to induce cell death in leukemic cells may be a viable strategy for future therapies, as some potential anti-cancer drugs act via the eIF2/ATF4 signaling pathway, which in turn promotes apoptosis [52–59].

Besides inducing apoptosis, PERK-mediated eIF2α phosphorylation can also induce autophagy [22, 23]. Accordingly, our data show that deletion of NLRP3, resulting in enhanced PERK/eIF2α activation, boosts autophagic flux in human AML cells. In support of our findings, Zhang et al. found increased autophagy levels at baseline and under hypoxia in lungs of NLRP3 knockout mice [60]. In contrast, Deng et al. showed that overexpression of NLRP3 inflammasome-related proteins resulted in increased autophagy and LC3-II conversion in human macrophages [61] and Allaeys et al. reported that silencing of NLRP3 reduced autophagic activity in human osteoblasts [62]. The different models used to investigate how NLRP3 modulates autophagy may help to explain the observed discrepancies. However, it is well known that increased autophagy inhibits NLRP3 inflammasome activation by eliminating inflammasome components [29]. In AML, the role of autophagy remains poorly understood. While some studies indicate that increased autophagic flux benefits the survival of AML cells [24–26], others claim that loss of autophagy is harmful to AML patients [63]. Thus, autophagy may be a double-edged sword in AML. However, whether autophagy is beneficial or detrimental to AML cells may depend on the pathogenicity of AML subtypes and the associated mutations [27].

Finally, we investigated the role of NLRP3 in AML in vivo by engrafting NSG-S mice with human MOLM-13 ctrl and ΔNLRP3 cells. We showed that the reduced engraftment potential of ΔNLRP3 leukemia cells may be attributed to impaired in vivo survival of those cells without cell proliferation being affecting. Thus, the increased susceptibility of MOLM-13 ΔNLRP3 cells to undergo apoptosis may explain the reduced percentage of human CD45^+^ cells, representing the engrafted MOLM-13 cells, in the analyzed organs. Furthermore, we found that MOLM-13 cells which expressed high levels of NLRP3 induced remarkable clinical signs in immune-deficient mice. In contrast, injection of MOLM-13 ∆NLRP3 cells resulted in a less severe pathological phenotype, highlighting the critical role of NLRP3 as a novel driver of disease progression in AML. These findings are in line with recent observations by Zhong et al., who demonstrated that upregulation of NLRP3 expression in the murine AML cell line C1498 leads to increased expansion of leukemic cells in the BM, liver, and spleen, and shortens survival of lethally irradiated C57BL/6J recipient mice [10]. In addition, Hamarsheh et al. showed that *KRAS* mutations promoted NLRP3 inflammasome activation in C57BL/6 mice, while inhibition of NLRP3 inflammasome activation with MCC950 reduced myeloproliferation [9], further supporting that NLRP3 may act as a driver of AML progression in vivo.

## Conclusion

In conclusion, our study identifies the NLRP3/PERK/eIF2 axis as a novel driving force in AML and provides evidence that targeting NLRP3 induces apoptosis and autophagy through PERK-mediated eIF2α phosphorylation without impacting AML cell proliferation. While additional studies on the interplay between NLRP3, ER stress, and consequently eIF2 phosphorylation are necessary, our results indicate that targeting NLRP3 in AML patients may provoke ER stress, phosphorylation of eIF2α, and consequently apoptosis of AML blasts, thus providing a potential novel therapeutic strategy.

### Electronic supplementary material

Below is the link to the electronic supplementary material.

Supplementary Material 1

## Data Availability

The data presented in the study are deposited in the PRIDE repository (https://www.ebi.ac.uk/pride/archive), accession number PXD047745.

## Abbreviations

AIM2: Absent in melanoma 2
AML: Acute myeloid leukemia
ASC: Apoptosis-associated speck-like protein containing a caspase recruitment domain
ATF4: Activating Transcription Factor 4
ATP: Adenosine 5′-triphosphate disodium salt hydrate
Bax: Bcl-2-associated X protein
Bcl-2: B-cell lymphoma 2
BID: BH3 interacting-domain death agonist
BiP: Binding Immunoglobulin Protein
BM: Bone marrow
BM-MNC: Bone marrow mononuclear cells
Caspase 1: cysteine-aspartic acid proteas
CFSE: Carboxyfluorescein succinimidyl ester
CHOP: C/EBP Homologous Protein
CRISPR/Cas9: Clustered Regularly Interspaced Short Palindromic Repeats / CRISPR associated protein 9
ctrl: CRISPR/Cas9 control
eIF2: Eukaryotic Initiation Factor 2
ELISA: Enzyme-linked Immunosorbent Assay
ER: Endoplasmic reticulum
GCN2: General control nonderepressible 2
HC: Healthy controls
HGF: Hepatocyte growth factor
HPLC: High-performance liquid chromatography
HRI: Heme-regulated inhibitor
IL-18: Interleukin 18
IL-1b: Interleukin 1-beta
KRAS: Kirsten rat sarcoma virus
LC3BI/II: Microtubule-associated proteins 1 A/1B light chain 3B
LPS: Lipopolysaccharide
MS: mass spectrometry
NLRP1/3/7: Nucleotide-binding oligomerization domain, Leucine rich Repeat and Pyrin domain containing
NSG-S mice: NOD.Cg-Prkdcscid Il2rgtm1Wjl Tg(CMV-IL3,CSF2,KITLG)1Eav/MloySzJ
PB: Peripheral blood
PERK: Protein Kinase RNA-Like ER Kinase
PIGF-1: Placental growth factor
PKR: Protein kinase R
Puma: p53 upregulated modulator of apoptosis
RAC1 GTPase: Ras-related C3 botulinum toxin substrate 1 guanosine triphosphatase
ROS: Reactive oxygen species
TEM: Transmission electron microscopy
TNFα: Tumor necrosis factor alpha
wt: Wildtype
ΔNLRP3: CRISPR/Cas9 NLRP3 knockout
