# Supplementary material for: Targeting NLRP3 inhibits AML progression by inducing PERK/eIF2-mediated apoptosis
Source: Cell Commun Signal. 2024 Sep 2;22:424. doi: 10.1186/s12964-024-01777-6 (PMC11367831; doi:10.1186/s12964-024-01777-6)
Supplement: Supplementary file 1 — Supplementary Material 1 [file 12964_2024_1777_MOESM1_ESM.docx]

**Supplementary Figure**
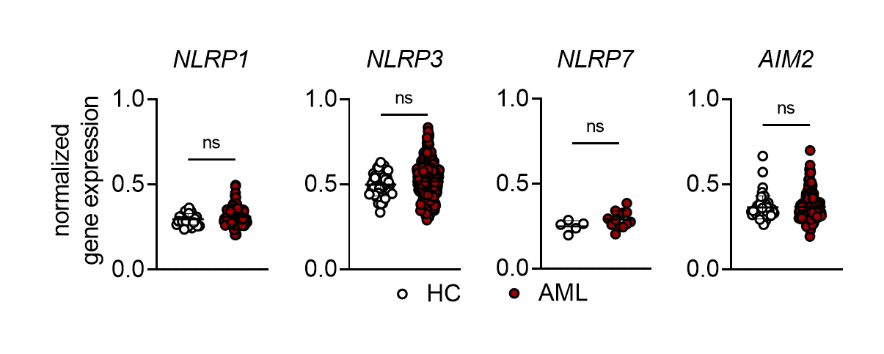
**s**

**Supplementary Figure 1: Expression of other NLR family members in AML patients**

*NLRP1*, *NLRP3, NLRP7,* and *AIM2* gene expression in AML patients (AML, n=542) compared to HC (n=72). Data were from the publicly available dataset GSE13159. A Mann-Whitney U test was used for comparing two groups. Data are shown as mean ± SD, ns, not significant.


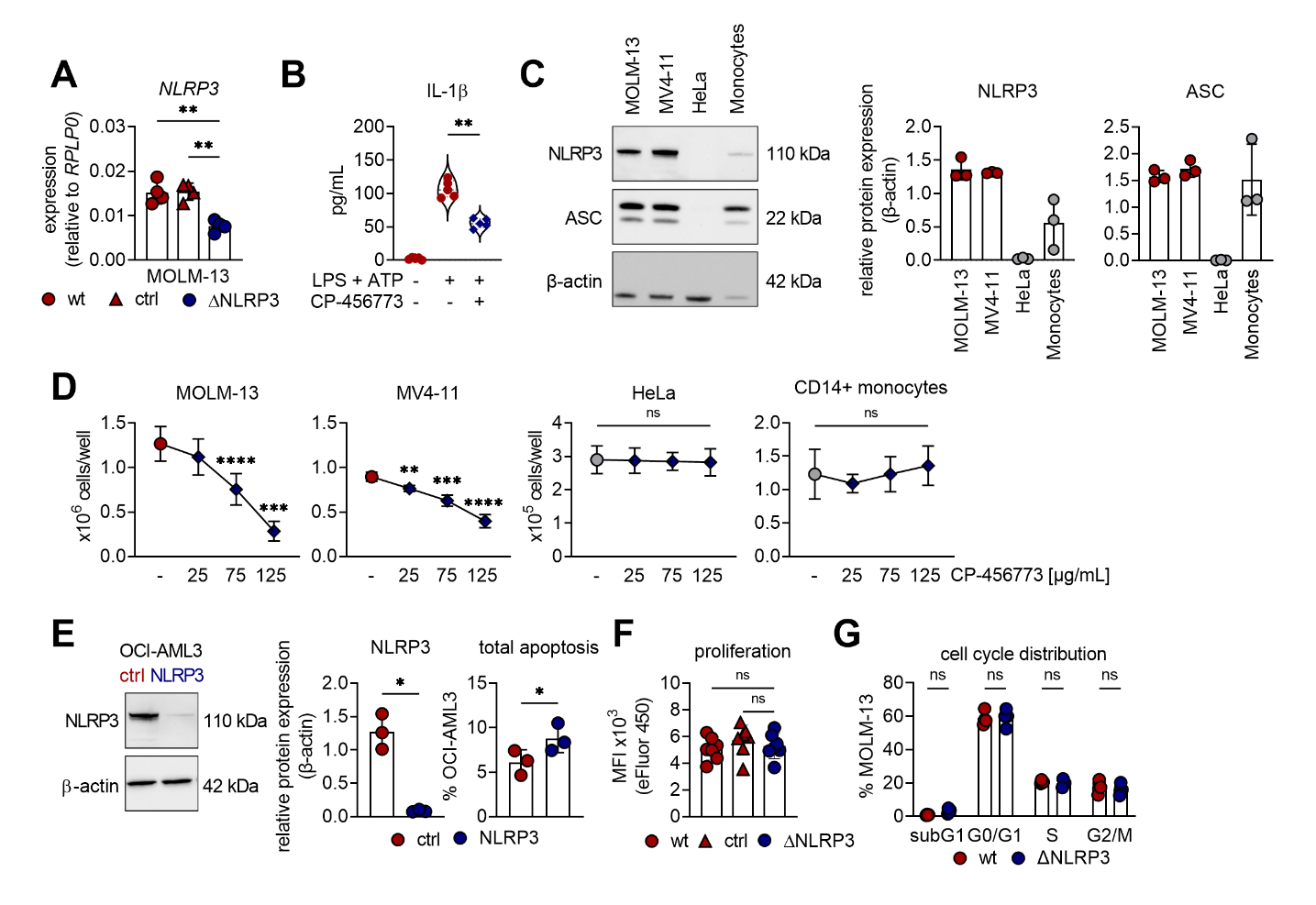
**Supplementary Figure 2: Effects of NLRP3 inhibition in different cell lines.**

**A** *NLRP3* mRNA levels were determined in MOLM-13 wild-type (wt), CRISPR/Cas9 control off-NLRP3-target (ctrl) and CRISPR/Cas9 NLRP3 knockout (ΔNLRP3) cells by qRT-PCR (n=4, relative mRNA expression to the housekeeping gene RPLP0). **B** MOLM-13 wt cells were stimulated with *E. coli* lipopolysaccharide (LPS, 50 ng/mL) for 4 h and then treated with CP-456773. After 2 h of NLRP3 inhibition, cells were stimulated with adenosine triphosphate (ATP, 5 mM) for 16 h to induce NLRP3 inflammasome activation. Supernatants were collected and analyzed for IL-1β secretion by ELISA (n=5). **C** NLRP3 and ASC expression was detected in the human AML cell lines MOLM-13 and MV4-11, the cervical cancer cells (HeLa) and CD14^+^ primary human monocytes by Western blot. One representative immunoblot out of 3 is shown. β-actin was used as a loading control for the densiometric quantification of NLRP3 and ASC protein levels. **D** Cell counts of MOLM-13, MV4-11, HeLa cells and CD14^+^ monocytes upon treatment with the indicated concentrations of CP-456773 for 72 h were evaluated using a Neubauer chamber (n=3-5). Asterisks indicate significant differences compared to untreated cells. Mean ± SD is shown. **E** OCI-AML3 were transiently silenced with a non-targeting control (ctrl) and NLRP3 targeting siRNA. NLRP3 and β-actin were detected 48 hours post transfection by Western blot analysis. β-actin was used as a loading control for the densiometric quantification of NLRP3. Bar chart showing the percentage of apoptotic OCI-AML3 cells after transient silencing with the ctrl and NLRP3 siRNA 48 hours post transfection (n=3). **F** Proliferation of MOLM-13 wt, ctrl, and ΔNLRP3 cells (n=7) was monitored 48 h after seeding by flow cytometry (n=7) by monitoring dye dilution of eFluor450. **G** Bar diagram showing cell cycle profiles of MOLM-13 wt and ΔNLRP3 cells 48 h after seeding (n=3). Dots in graphs indicate individual replicates; bars represent mean ± SD. A two-tailed, paired t-test (**E**) was used for comparing two groups, a one-way ANOVA with Tukey’s post-hoc test (**A, B, F**) was performed for multiple comparisons, a two-way ANOVA with Dunnett’s post-hoc test (**D**) or Šídák's post-hoc (**G**) was performed for multiple comparisons. Significance levels are defined as follows: *, p ≤ 0.05; **, p ≤ 0.01; ***, p ≤ 0.001; ****, p ≤ 0.0001; ns, not significant.


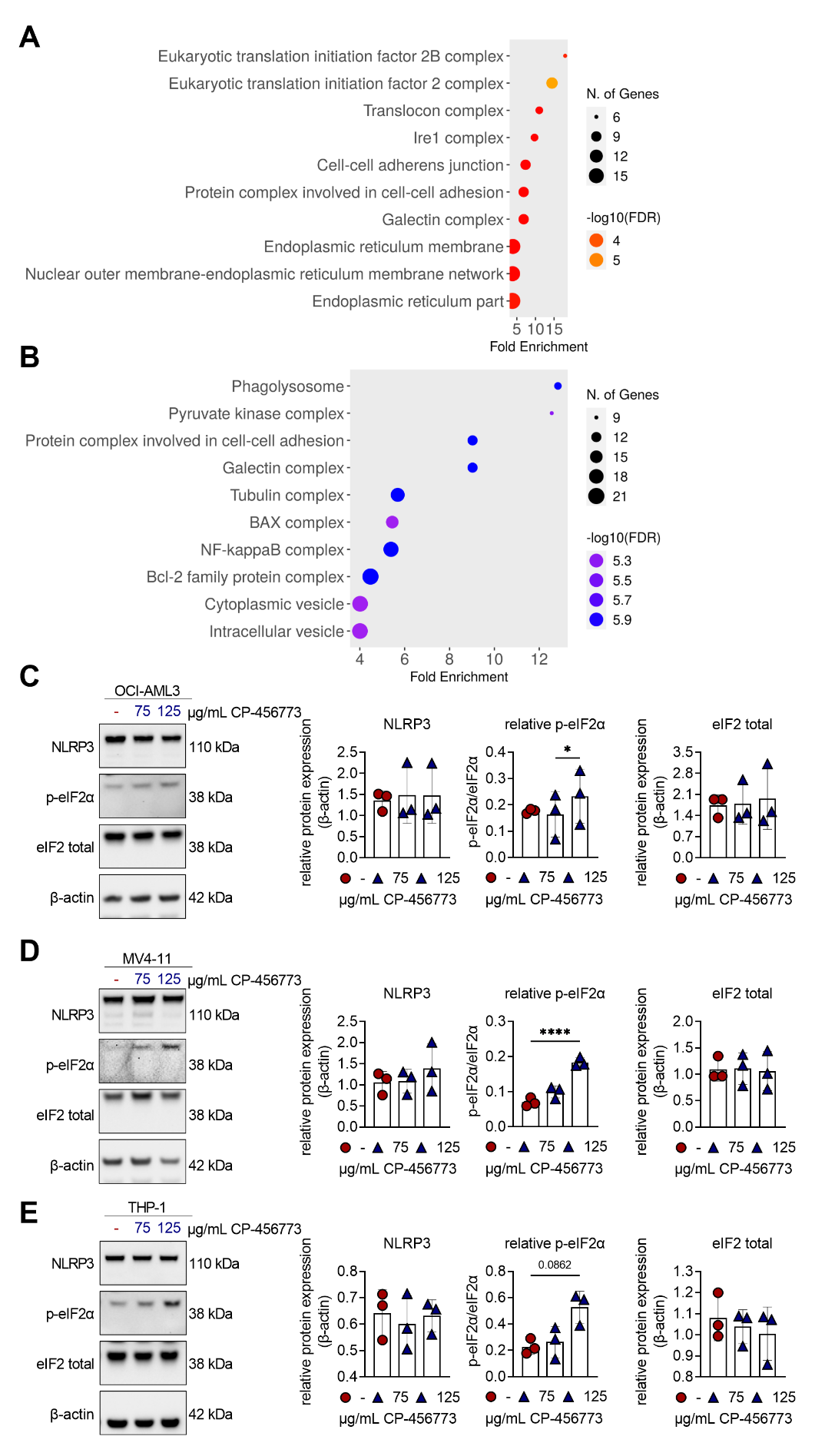
**Supplementary Figure 3: Targeting NLRP3 induces enrichment of the EIF2 complex and ER related pathways.**

**A/B** Pathway and complex analysis of the top 100 significantly up- **(A)** and downregulated **(B)** proteins was performed using ShinyGO (1). ). **C-E** OCI-AML3 (**C**), MV4-11 (**D**) and THP-1 (**E**) cells were either left untreated (-) or treated with 75 or 125 µg/mL CP-456773 for 24 h before being processed for Western blot analysis of NLRP3, p-eIF2α, eIF2 total and β-actin (n=3). The ratio of phosphorylated to total eIF2 was calculated. β-actin was used as a loading control for the densiometric quantification of NLRP3 and eIF2 total. One-way ANOVA with Tukey’s post-hoc test (**C-E**) was performed for multiple comparisons. Significance levels are defined as follows: *, p ≤ 0.05; ****, p ≤ 0.0001.


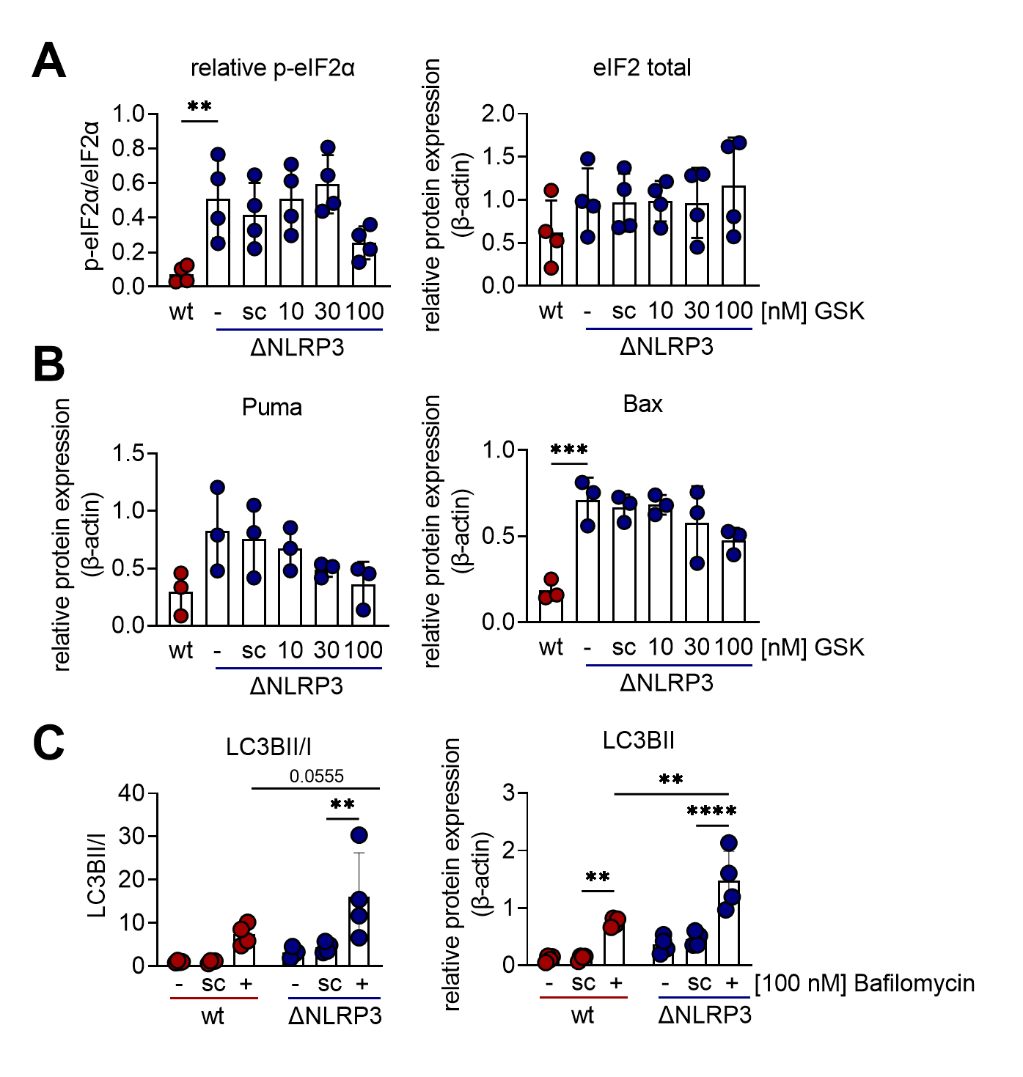
**Supplementary Figure 4: PERK inhibition reduces phosphorylation of eIF2α and expression of pro-apoptotic proteins.**

**A-C** Densiometric quantifications of Western blots shown in Fig 4B, 4E and 4I. **A** Phosphorylated eIF2α relative to eIF2 total and eIF2 total relative to β-actin (n=4). **B** Puma and Bax relative to β-actin (n=3). **C** Densiometric quantification of LC3BII relative to LC3BI and LCB3II relative to β-actin (n=4). One-way ANOVA with Šídák's post-hoc test (**A-C**) was performed for multiple comparisons. Significance levels are defined as follows: **, p ≤ 0.01; ***, p ≤ 0.001; ****, p ≤ 0.0001.


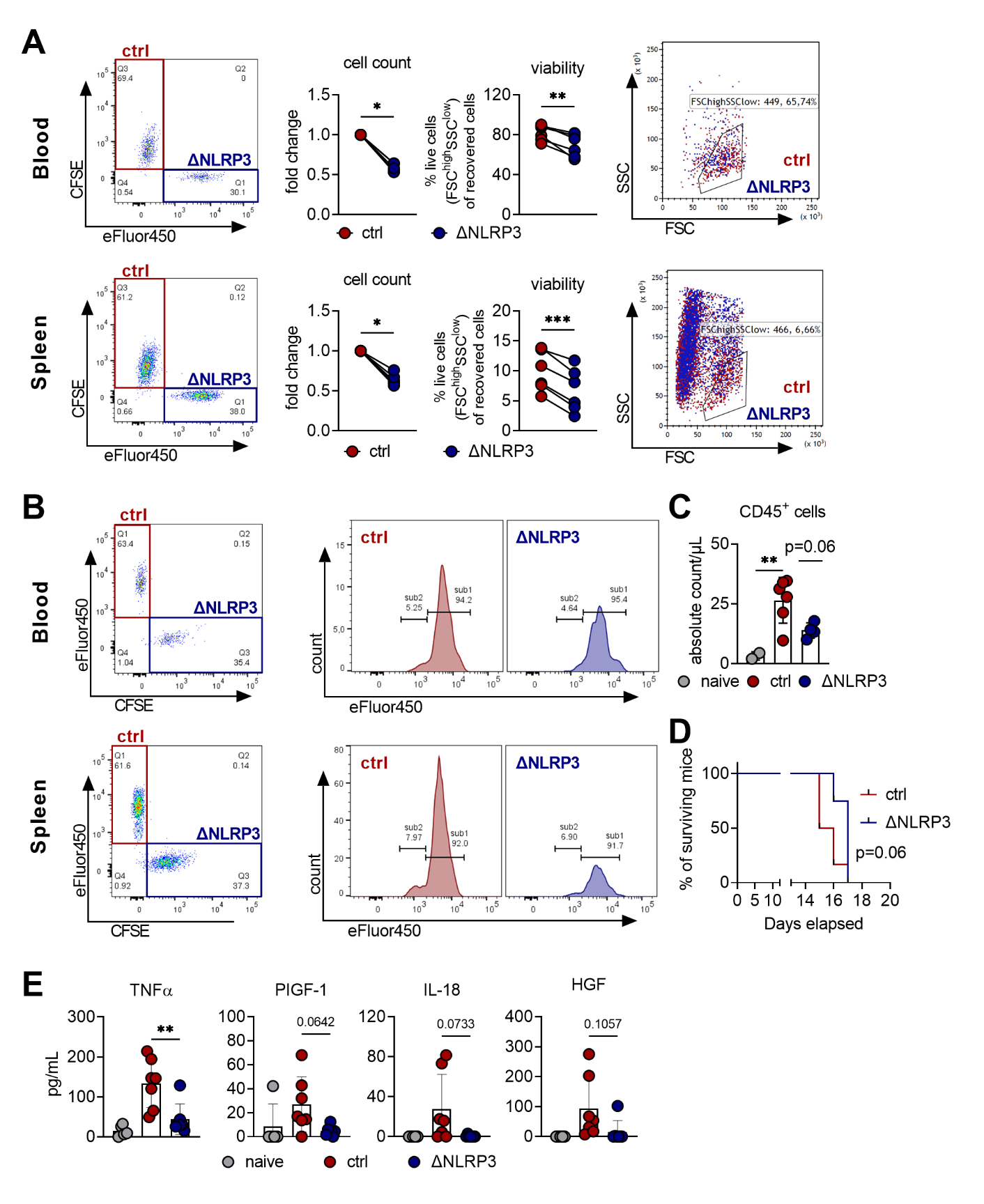


**Supplementary Figure 5: NLRP3 is required for homing and survival in vivo.**

**A** Flow cytometric analysis of relative cell counts and cell viability, as determined by FSC^high^SSC^low^ gating strategy, of CFSE- or eFluor450- labeled ΔNLRP3 cells or ctrl cells in the PB and spleen of NSG-S mice after 48 h. **B** Flow cytometry plots of eFluor450-labeled ctrl cells and CFSE-labeled ΔNLRP3 cells in the PB and spleen of NSG-S mice 48 h after cell injection. Proliferation of ctrl cells and ΔNLRP3 cells in the PB and spleen of NSG-S mice 48 h post cell injection. For both ctrl and ΔNLRP3 samples, n = 6**. C** Absolute CD45^+^ cell counts per µL blood were measured 11 days post injection. The blood sample was taken from the tail vein. NSG-S mice that received PBS served as a non-leukemic control (=naïve). **D** Kaplan-Meyer survival curve of NSG-S mice engrafted with MOLM-13 ctrl or ΔNLRP3 cells. Naïve samples, n=2; ctrl samples, n=6; ΔNLRP3 samples, n=4. **E** Serum levels of human-derived pro-inflammatory cytokines and growth factors of naïve, ctrl and ΔNLRP3 mice. Naïve samples, n= 5; ctrl samples, n= 7; ΔNLRP3 samples, n= 7. Data were tested for normality and appropriate statistical tests were used: Statistical analyses were performed by Wilcoxon matched-pairs signed rank test (**A**: cell count), a paired t-test for the analysis between two groups (**A**: viability) or one-way ANOVA with Tukey’s post-hoc test was performed for multiple comparisons (**C+E**), while a Cox proportional hazards model was used for the Kaplan-Meier curve (**D**). Significance levels are defined as follows: *, p ≤ 0.05; **, p ≤ 0.01; ***, p ≤ 0.001; ns, not significant.

**Supplementary materials and methods**

**Database analysis**

Public genome datasets GSE13159 and GSE12417 from NCBI’s Gene Expression Omnibus (NCBI-GEO) were used. Based on the GSE13159 dataset, *IL1B, IL18, ASC,* and *NLRP3* expression was evaluated in AML patients and in healthy individuals of bone marrow samples. The dataset used is part of the MILE (Microarray Innovations in Leukemia) study research program. The study includes whole-genome analysis data from 542 AML patients, 76 CML patients, and 74 healthy donors for a total sample size of 2096 from 11 participating centers on three continents (2, 3). Gene expressions were determined by hybridizing blood or bone marrow samples to Affymetrix HG-U133 Plus 2.0 GeneChips. For bioinformatics analysis we used the non-central trimmed mean of differences between perfect match (PM) and mismatch (MM) intensities with quantile normalization (DQN) signals (4). Only gene expression signals from bone marrow samples with significant detection p-value (p < 0.05) were used. The GSE12417 dataset was used to analyze NLRP3 expression among AML French-American-British (FAB) subclasses (5), which includes gene expression profiling data of 163 patients treated in the German AMLCG 1999 trial (6-thioguanine/cytarabine/daunorubicin and/or high-dose cytarabine/mitoxantrone regimens) to predict the overall survival (OS) in cytogenetically normal AML (CN-AML) (6). Datasets from the GEO database were imported using GEOparse (https://geoparse.readthedocs.io/) and the analysis was performed using Python.

**Primary human AML samples, cell lines, and culture conditions**

All studies involving human cells were conducted in accordance with the guidelines of the World Medical Association’s Declaration of Helsinki.

All cells were cultured at 37 °C, 5 % CO_2_ in a humified atmosphere. Following written informed consent, BM aspirate samples were collected from patients with newly diagnosed AML at Paracelsus Medical University Hospital, Salzburg, Austria (Ethics committee Salzburg approval: 415-E/2009/2-2016). BM samples from healthy donors were obtained from Caltag Medsystems, UK (https://www.caltagmedsystems.co.uk/). Human BM mononuclear cells (BM-MNC), CD34^+^ umbilical cord, and CD34^+^ hematopoietic stem/progenitor cells (Ethics Committee Salzburg approval 415-E/1776/4-2014) were isolated using density gradient centrifugation (Lymphoprep; Stemcell Technologies). Human primary monocytes were isolated and cultured as described previously (7). Briefly, monocytes were isolated from fresh buffy coats of healthy volunteers obtained from the Blood Bank Salzburg. Gradient density centrifugation using HistoPaque-1077 (Sigma-Aldrich, Catalog number: 10771) was used to isolate peripheral blood mononuclear cells (PBMCs). Magnetic labeling with CD14 MicroBeads (Miltenyi Biotec, Catalog number: 130-050-201) was employed to purify the monocytes according to the manufacturer’s instructions. Thereafter, monocytes were cultured in RPMI-1640 medium (Sigma-Aldrich, Catalog number: R0883) supplemented with 10% heat-inactivated fetal bovine serum (FBS; Catus Biotech, Catalog number: BS-2020-500) and 2 mM L-glutamine (Sigma-Aldrich, Catalog number: G7513). Primary patient cells, MOLM-13, MV4-11, OCIAML3 and THP1 cells (purchased from Leibniz-Institut DSMZ GmbH) were cultured in RPMI-1640 medium supplemented with 10 % heat-inactivated FBS, 1 % penicillin and streptomycin (Sigma-Aldrich, Catalog number: P4333), and 2 mM L-glutamine. The human cervix carcinoma cell line (HeLa) was cultured in Minimum Essential Medium Eagle (MEM; Sigma-Aldrich, Catalog number: M2279) supplemented with 10 % heat-inactivated FBS, 1 % penicillin and 1 % streptomycin, 2 mM L-glutamine and 2 mM non-essential amino acids (Sigma-Aldrich, Catalog number: M7145). All cell lines were routinely screened for mycoplasma contamination (MycoAlert™ PLUS Mycoplasma Detection Kit; Lonza, Catalog Number: LT07-705) following the instructions of the manufacturer.

2x10^5^ AML cells/mL were seeded in appropriate cell culture plates and incubated for the indicated time points. 4x10^4^ HeLa cells were seeded per well in a 24 well plate and incubated for the indicated time points.

For NLRP3 inhibition, the small molecule CP-456773 sodium salt ≥ 98 % (HPLC) (Sigma-Aldrich, Catalog number: PZ0280) was used. The PERK inhibitor GSK2606414 was purchased from Selleckchem (Selleckchem, Catalog number: S7307) and Bafilomycin A1 was obtained from Sigma (Sigma-Aldrich, Catalog number: SML1661).

**Generation of NLRP3-deficient MOLM-13 cells (MOLM-13 ΔNLRP3)**

To generate non-targeting control (ctrl) or NLRP3 knockout (∆NLRP3) MOLM-13 cells, lentiviral particles (0.5 MOI) produced in HEK293FT cells transiently transfected with two packaging plasmids (psPAX2 and pMD2-G) and the lentiCRISPR v2 plasmid (Addgene, #52961) (8) containing a non-targeting (GGCATCTTAACTAATCGTCT) or NLRP3-targeting (AAAAGAGATGAGCCGAAGTG; CRISPick design tool (9, 10)) sgRNA were used to transduce MOLM-13 cells via spin-infection (11). Successfully transduced cells were selected for puromycin resistance. NLRP3 protein knockout validation was performed by Western blot analysis.

**Immunohistochemical staining and detection of cytokines**

Immunohistochemistry (IHC) for NLRP3/NALP3 (Adipogen Life Sciences, AG-20-b-0014-C100, Liestal, Swiss) was performed on prepared cell blocks of the human cancer cell lines MOLM-13 and MOLM-13 ΔNLRP3 as well as on routinely archived formalin-fixed paraffin-embedded (FFPE) BM trephine samples of normal controls (n= 10) and AML cases (n= 13, initial diagnosis n= 11) (AML-M5, NOS according to WHO classification). Sections were stained with primary NLRP3/NALP3 antibody using a Benchmark Ultra platform (Ventana, Tucson, USA) and an ultraView Universal DAB Detection Kit (Ventana) after application of the amplification kit (Ventana). The results were scored by assessing the extensity (% positive cells) and intensity of IHC staining (0-3) on three different representative microscope fields and expressed semi-quantitatively using the quickscore method by multiplication of the extensity and intensity (yielding values between 0-300) for each field (12). Analysis of cytokine secretion in primary BM-MNCs from AML patients and sera obtained from mice engrafted with MOLM-13 ctrl and MOLM-13 ΔNLRP3 cells was performed using the Cytokine/Chemokine/Growth Factor 45-Plex Human ProcartaPlex™ system (Invitrogen, Catalog number: EPX450-12171-901).

**Inflammasome activation and ELISA**

CP-456773 sodium salt ≥ 98 % (HPLC) (Sigma, Catalog number: PZ0280) was dissolved according to the manufacturer’s instructions. MOLM-13 cells were seeded in a 48-well plate at a density of 2×10^5^/mL. To assess the functionality of CP-456773, the cells were stimulated with 50 ng/mL LPS from *Escherichia coli* 055:B5 (Sigma, Catalog number: L2880-10MG) for 4 h and then treated with the NLRP3 inhibitor CP-4546773 (125 µg/mL). 2 hours later, cells were stimulated with 5 mM adenosine 5′-triphosphate disodium salt hydrate (ATP) for 16 h (InvivoGen, Catalog number: tlrl-atpl). Cells were properly resuspended, transferred into 1.5 mL tubes and spun down. The supernatants were harvested and analyzed for IL-1β using commercially available ELISA kits (R&D Systems, Catalog number: DY202), according to the manufacturer’s instructions.

**qRT-PCR**

Pellets of cultured cells were directly lysed in Trizol (Sigma, Tri Reagent®, Catalog number: T9424) and RNA was isolated according to the manufacturer’s instructions. Complementary DNA (cDNA) was generated using RevertAid H Minus M-MulV reverse transcriptase (Thermo Fisher Scientific, Catalog number: EP0451). Expression levels were determined by quantitative real-time PCR on a Rotorgene 3000 (Qiagen Instruments) using Luna® Universal Probe qPCR Master Mix (New England BioLabs® Inc, Catalog number: M3003). The large ribosomal protein P0 (RPLP0) was used as a reference gene. Relative mRNA expression (x) was calculated as x = 2^-ΔCt^, where Δct represents the difference between the threshold cycle (ct) of the target gene and the reference gene. NLRP3 primer pair (Sigma-Aldrich): forward 5' -TCAGCACTAATCAGAATCTCACGCACCTTT -3' and reverse 5' -CCAGGTCATTGTTGCCCAGGCTC -3'; CHOP primer pair (Sigma-Aldrich): forward 5’ -CAAGAGGTCCTGTCTTCAGATGA -3’ and reverse 5’-TCTGTTTCCGTTTCCTGGTCC-3’; RPLP0 primer pair: forward 5' -GGCACCATTGAAATCCTGAGTGATGTG -3' and reverse 5' -TTGCGGACACCCTCCAGGAAG -3'.

**Western blot**

Pellets of cultured cells were lysed in 80 µL of 2x Laemmli sample buffer (BIO-RAD, Catalog number: 1610737) with 5 % β-mercaptoethanol (Sigma-Aldrich, Catalog number: M6250). Samples were separated on 4 % to 12 % gradient gels (NuPAGE, Life Technologies, Catalog number: NP0321) and subsequently blotted onto a 0.45 µM nitrocellulose membrane (BIO-RAD, Catalog number: 1620115) using a semi-dry transfer system. The membranes were blocked with 5 % nonfat dry milk in 1x TBS containing 0.1 % Tween20. All antibodies were purchased from Cell Signaling and used according to the manufacturer’s instructions: NLRP3 (15101), ASC (13833), BID (2002), Puma (12450), Bax (5023), eIF2α (5324), phospho eIF2α (Ser51) (3398), LC3A/B (12741), β-actin (4970) and HRP-linked anti-rabbit secondary antibody (7074). Chemiluminescent detection was used (Thermo Fisher, Super Signal West Pico Plus Chemiluminescence, Catalog number: 34580). ImageJ (NIH) was used for the quantification of Western Blots.

**Cell proliferation, cell cycle and apoptosis analyses**

MOLM-13 wt, ctrl and ΔNLRP3 cells were seeded in 24- or 48-well plates at a density 2×10^5^ cells per mL and harvested according to manufacturer’s instructions for the chosen readout. For cell counting assays, 500 µL AML cells were seeded in 48-well plates at a density of 2×10^5^ cells per mL and harvested by proper resuspension in the well for manual cell counting using a Neubauer counting chamber (cells were diluted 1:2 with Trypan blue) after the indicated time points. In contrast, 4x10^4^ HeLa cells were seeded in 24 well plates to prevent early confluency. Adherent cells were harvested using 1x Trypsin-EDTA to detach the cells: first, the supernatant was removed, and 1 mL PBS was added for washing and immediately removed again. 200 µL 1x Trypsin-EDTA was added and incubated at 37°C for 3 minutes. Next, 800 µL fully supplemented MEM medium was added to stop the trypsinization process. Thereafter, HeLa cells were detached and counted using the Neubauer counting chamber as described for AML cells.

For proliferation analyses, 1×10^7^ cells were stained with 2 µM of the proliferation dye (Invitrogen, eBioscience™ Cell Proliferation Dye eFluor™ 450, Catalog number: 65-0842-85) diluted in PBS. Cells were washed with complete RPMI medium and seeded in 48-well plates at a density of 2×10^5^ cells per mL and cultured for 48 h. Proliferation (= median fluorescence intensity of eFluor450) was assayed by flow cytometry by recording events for 120 seconds. Viable cells were identified using the fixable viability dye eFluor™ 780 (Invitrogen, eBioscience™ Fixable Viability Dye eFluor™ 780, Catalog number: 65-0865-18). Freshly eFluor450 stained cells were directly measured after staining, serving as a negative control. For cell cycle analysis cells were stained with the FxCycle™ PI/RNase Staining Solution according to the manufacturer´s instructions (Invitrogen, Catalog number: F10797). Viable cells were identified using the fixable viability dye eFluor™ 780 (Invitrogen, eBioscience™ Fixable Viability Dye eFluor™ 780, Catalog number: 65-0865-18). 20.000 events were recorded to analyze the cell cycle distribution.

The rate of apoptosis was measured using an Annexin V Apoptosis Detection Kit according to the manufacturer´s instructions (Invitrogen, eBioscience™ Annexin V Apoptosis Detection Kits, Catalog number: 88-8006-74). For this purpose, 2x10^5^ cells per mL were seeded and incubated for 48 h. Viable cells are represented by the percentage of Annexin V^-^/7-AAD^-^ cells, while apoptotic cells are shown as the sum of Annexin V^+^/7-AAD^-^ and Annexin V^+^/7-AAD^+^ cells. Flow cytometric data were detected with a BD FACS Canto II and analyzed using FlowJo software (FlowJo v10.7.1, BD Biosciences).

**siRNA-based silencing in AML cells**

For transient transfections, OCI-AML3 cells were transfected with a mix of three small interfering RNAs directed against NLRP3 (Thermo Fisher scientific, Catalog number: HSS132811, HSS132812, HSS132813) or Allstars negative Control (Qiagen, Catalog number: 1027281) as described previously (13). Briefly, 2x10^5^ cells were seeded in 400 µL Opti-MEM (Gibco™, Opti-MEM™ I Reduced Serum Medium, Catalog number: 31985-047) and transfected with 100 pmol of the siRNA mix and 1.5 µL Lipofectamine RNAiMax (Invitrogen, Lipofectamine™ RNAiMAX Transfection Reagent, Catalog number: 13778030) in 100 µL Opti-MEM. After 6 hours of incubation, 500 µL of fresh complete RPMI-1640 were added and cells were incubated for 48 hours before being processed for Western blot analysis and flow cytometry to determine the percentage of apoptotic cells.

**Animal studies**

Male 10- to 16-week-old NOD.Cg-Prkdc^scid^ Il2rg^tm1Wjl^ Tg(CMV-IL3,CSF2,KITLG)1Eav/MloySzJ (NSG-S) mice obtained from the Jackson Laboratories were kept under specific pathogen free conditions, in a 12-h light-dark cycle, with a standard chow diet and water ad libitum at the animal facility of the University of Salzburg. All mouse experiments were approved by the Federal Ministry of Education, Science and Research (BMBWF), Austria (permission number: BMWFW-66.012/0032-WF/V/3b/2017 and 2023-588.057) and complied with EU guidelines (2010/63/EU) and Austrian law (TVG 2012).

For testing the behavior of MOLM-13 cells in vivo, MOLM-13 control cells or ∆NLRP3 cells were stained with the cell trackers Carboxyfluorescein succinimidyl ester (CFSE; Invitrogen™, Catalog number: C34554) or Cell Proliferation Dye eFluor 450 (Invitrogen™, Catalog number: 65-0842-85), mixed and 10×10^6^ cells were then injected intravenously into NSG-S mice. For quantification of stained cells, bone marrow, spleen, and peripheral blood of recipients were harvested 48 h post injection. Bone marrow- and spleen-derived single-cell suspensions were obtained by mechanically disrupting the organs and filtrating cells through a 40 µm mesh. Red blood cells were removed by ACK lysis buffer incubation. Human CD45^+^ (CD45-PerCP-Cy5.5, Biolegend, Catalog number: 304028) and CFSE^+^ or eFluor450^+^ cells were detected by flow cytometry (BD FACS Canto II) using Precision Count Beads™ (BioLegend, Catalog number: 424902) according to the manufacturer’s instructions and analyzed using FlowJo software (FlowJo v10.7.1, BD Biosciences).

For the MOLM-13 tumor engraftment studies, NSG-S mice were intravenously injected with 0.5 × 10^6^ MOLM-13 ctrl or 0.5 × 10^6^ ∆NLRP3 cells and constantly monitored and scored for leukemia-associated changes in physical appearance, breathing rate and behavior. The experiment was repeated twice. All mice of the same experiment were euthanized when predefined termination criteria were met, which was at day 18 after injection in the first experiment, and on day 14 in the second experiment. Peripheral blood, spleen and bone marrow were isolated and evaluated for the percentage of human CD45^+^ cells by flow cytometry (CD45-PerCP-Cy5.5, Biolegend, Catalog number: 304028). Flow cytometric data were detected with a BD FACS Canto II and populations were defined and analyzed using the FlowJo software (Flowjo v10.7.1, BD Biosciences).

For the Kaplan Meyer curve, 24 weeks old female and male NSG-S mice were intravenously injected with 0.5 × 10^6^ MOLM-13 ctrl or 0.5 × 10^6^ ∆NLRP3 cells and constantly monitored and scored as described above. The mice were euthanized between 14 and 18 days after injection. The experiment was conducted once. 11 days post injection, 5 µL blood were taken from the tail vein and absolute numbers of human CD45^+^ (CD45-PerCP-Cy5.5, Biolegend, Catalog number: 304028) cells were detected by flow cytometry (BD FACS Canto II) using Precision Count Beads™ (BioLegend, Catalog number: 424902) according to the manufacturer’s instructions.

**Proteomics**

**Chemicals.** Dithiothreitol (DTT, ≥ 99.5 %), formic acid (FA, 98.0-100 %), iodoacetamide (IAA, ≥ 99.0 %), sodium dodecyl sulfate (SDS, ≥ 99.5 %) and triethylammonium bicarbonate (TEAB, pH 8.5, 1 mol/L) were obtained from Sigma-Aldrich (Vienna, Austria). Acetonitrile (ACN, ≥ 99.9 %) and methanol (MeOH, ≥ 99.9 %) were obtained from VWR International (Vienna, Austria). Ammonia (25 %) and ortho-phosphoric acid (85 %) were purchased from Merck (Burlington, MA, USA). Trypsin (sequencing grade modified, porcine) was obtained from Promega (Madison, WI, USA). A MilliQ Integral 3 instrument (Millipore, Billerica, MA, USA) was used for deionization of water.

**Cell culture.** MOLM-13 ctrl and ΔNLRP3 cells were seeded in a 48-well plate at a density of 2×10^5^/mL and incubated for 24 h at 37°C and 5 % CO2. Biological replicates were generated by conducting this treatment scheme on three consecutive days. Cells were washed thoroughly with PBS before sample preparation.

**Sample preparation.** S-Trap mini columns (Protifi, Huntington, NY, USA) were employed according to the manufacturer´s instructions with minor adjustments: a cell pellet of approximately 1×10^6^ cells was lysed in 5 % SDS and 50 mmol/L TEAB (pH 7.55) at 95 °C for 5 min followed by sonication in a Bioruptor device (Diagenode, Liège, Belgium) for 10 min. After a centrifugation step, protein content was analyzed by a Pierce BCA Protein assay kit (Thermo Fisher Scientific, Vienna, Austria). Denaturation and reduction of proteins were performed by supplementation of DTT to 40 mmol/L and incubation at 95 °C for 10 min. Reduced cysteine residues were alkylated by the addition of IAA to a concentration 80 mmol/L and incubation at 21 °C in the dark for 30 min. After a precipitation step and thorough washing, 10 µg of trypsin were solubilized in 50 mmol/L TEAB (pH 8.5), added to the S-Trap matrix and incubated at 37 °C for 18 h. Peptides were eluted and subsequently dried at 30 °C using a vacuum centrifuge. Samples were resuspended in 100 mmol/L TEAB (pH 8.5) to a concentration of 1.0 mg/mL. 100 µg of peptides of each sample were labeled by using a TMT 10plex™ kit (Thermo Fisher Scientific) according to the manufacturer´s instructions. Samples were labeled with the following tags: MOLM-13 ctrl (126, 128N, and 129C) and ΔNLRP3 (127C, 129N, and 130C). All six samples were pooled and dried at 30 °C in a vacuum centrifuge. The combined sample was resuspended in H2O + 0.1% formic acid to a concentration of 2.0 mg·mL-1.

**High-performance liquid chromatography and mass spectrometry.** Peptides were separated employing a 2000 mm µPAC™ C18 column (PharmaFluidics, Ghent, Belgium). These nanoscale chromatographic separations were carried out on a nanoHPLC instrument (UltiMate™ U3000 RSLCnano, Thermo Fisher Scientific, Germering, Germany) at a flow rate of 300 nL/min and a column oven temperature of 50 °C. Mobile phase solution A contained H2O + 0.10 % FA, and mobile phase B contained ACN + 0.10 % FA. After solvent B was kept at 1.0 % B for 5.0 min, a linear gradient to 40.0 % B in 595.0 min was applied. This gradient was followed by a purging step at 90.0 % B for 30.0 min. The column was re-equilibrated at 1.0 % B for 100.0 min. 1.0 µl of combined sample was injected using a microliter pick-up mode (5.0 µl loop volume). Three technical replicates were measured.

The nanoHPLC was hyphenated to a quadrupole-Orbitrap hybrid mass spectrometer (Thermo Scientific QExactive Plus benchtop quadrupole-Orbitrap mass spectrometer) via a Nanospray Flex ion source (both from Thermo Fisher Scientific, Bremen, Germany). The source was equipped with a SilicaTip emitter with 360 µm o.d., 20 µm i.d. and a tip i.d. of 10 µm purchased from New Objective (Woburn, MA, USA). The mass spectrometer was operated with the following instrument settings: spray voltage of 1.5 kV, S-lens RF level of 55.0, capillary temperature of 320 °C and an MS1 AGC target of 3e6 in an m/z range of 400-2000 with a maximum injection time of 100 ms. An MS1 scan at a resolution setting of 70,000 at 200 m/z was followed by 15 data-dependent MS2 scans at a resolution of 35,000 at m/z 200. Target peptides were fragmented by HCD at 32.0 NCE in a 2.0 m/z isolation window with an AGC target of 1e5 and a maximum injection time of 100 ms. A dynamic exclusion setting of 30 s was applied. Pierce LTQ Velos ESI Positive Ion Calibration Solution from Life Technologies (Vienna, Austria) was used for the calibration of the instrument.

**Data evaluation.** Acquired raw data were evaluated using MaxQuant software (v1.6.12.0) (14) in default settings correcting for isotope impurities in TMT reagents (provided by the manufacturer). Uniprot database entries (Swiss-Prot) for *Homo sapiens* (access: 30.03.2020) were provided for MaxQuant protein identification (15). Identified protein groups were imported into the R statistical programming language (v4.3.0). Groups tagged as potential contaminants or decoys were removed. All three biological replicates were required to have valid experimental measurements for both control and ΔNLRP3. Principal component analysis (PCA) of reporter intensity data with applied correction factors was carried out with package pcaMethods (16) after linearly scaling down measurements by a multiplicative factor of 10^-7^ to facilitate readability of the PCA plot. The same reporter intensities were imported into NormalyzerDE (17) web interface v1.14.0. The analysis was performed in two steps. First, we evaluated different reporter intensity normalization methods and in a second step normalized data were submitted to differential abundance analysis utilizing the Limma empirical Bayes method with default parameters (no RT-segmented normalization). The design was chosen such that abundance was calculated by contrasting ΔNLRP3 versus control, three replicates per condition. We adjusted for multiple hypothesis testing by employing Benjamini-Hochberg correction at a false discovery rate of 0.05. Gene set enrichment on proteins ordered by log fold change was computed for the canonical pathways dataset from the Molecular Signatures Database Human Collections (MSigDB 2023.1.Hs) (18) with R Bioconductor package fgsea (v1.26.0). Gene identifier mappings were retrieved from the HGNC database genenames.org (19) on July 13, 2023. Pathway and complex analysis of the top 100 significantly up- and downregulated proteins was performed using ShinyGO (1) with the following settings: false discovery rate 0.05; pathway size >2 and <2000; pathway database: Onotology.Jensen.COMPARTMENTS. Graphs were generated with ggplot2 (v3.4.2), ShinyGO, and GraphPad Prism 8 software (GraphPad Software, San Diego, CA, USA).

**Data availability.** The mass spectrometry proteomics data have been deposited in the ProteomeXchange Consortium via the PRIDE partner repository with the dataset identifier PXD047745 (20).

**Transmission electron microscopic (TEM) preparation**

MOLM-13 wt and ΔNLRP3 MOLM-13 cells were transferred to designated sample holders for high-pressure freeze fixation (HPF). The HPF process was carried out in a Leica Empact HPF device (Leica Microsystems, Vienna, Austria). A cooling rate of approx. 12.000 °C/s and a pressure value of at least 2040 bar were ensured for a sufficient HPF procedure. Subsequent cryosubstitution was performed in a Leica EM AFS (Leica Microsystems, Vienna, Austria). The following cycles were implemented: The HPF-fixed cells were first transferred to the pre-cooled substitution medium at -80 °C and kept there for 60 h. 2 % osmium tetroxide (OsO_4_) and 0.05 % uranyl acetate in anhydrous acetone were used as the substitution medium. The temperature was then further increased to -30 °C with + 10 °C/h. The samples were kept at this temperature for another 4 h. Finally, the temperature was increased with +2.5 °C/h and stabilized at +20 °C room temperature. In the next step, the samples were washed three times each with anhydrous acetone and propylene oxide and were then embedded in epoxy resin (medium quality; Agar Scientific, Essex, UK). Beem capsules (Agar Scientific, Essex, UK) were used for embedding to ensure cells settled at the narrow bottom of the capsules. This was of crucial importance for further sample preparation, especially for the following sectioning. The samples were then polymerized at +70 °C for 24 h. Finally, ultrathin sections (~70 nm) were cut on a Leica UC7 ultramicrotome (Leica Microsystems, Vienna, Austria) and were collected on Formvar-coated copper grids.

**2D Transmission electron microscopic (TEM) visualization**

2D TEM images of MOLM-13 wt and ΔNLRP3 MOLM-13 cells were acquired on a LEO 912 AB TEM with an in-column Omega energy filter (Zeiss, Oberkochen, Germany) at an acceleration voltage of 80 kV. Digital images were recorded with a bottom-mounted 2K CCD camera from Tröndle TRS Sharp Eye (Tröndle, Moorenweis, Germany). All TEM images were filtered at zero energy loss. iTEM 5.0 software (Olympus SIS, Münster, Germany) was used for TEM implementation and the recording process.

**Immunofluorescence and confocal microscopy**

Cytospin samples were generated by using a Cytospin 4 centrifuge (Epredia), where cells were directly spun onto a microscope slide at 400 rpm for 3 minutes. After short air-drying, sample regions were marked with a Hydrophobic Barrier Pap Pen (Thermo Fisher Scientific, Catalog number: R3777), and the cells were fixed with 4 % paraformaldehyde (, ThermoFisher Scientific, Image-iT, Catalog number: I28800) for 15 minutes at RT. Samples were transferred to a wet staining chamber and washed 3 times with PBS. Cells were permeabilized with 0.1 % TritonX100 (Sigma-Aldrich) for 10 min, followed by two washes with PBS. Unspecific binding sites were blocked with 2 % BSA and 5 % donkey serum (Sigma-Aldrich, Catalog number: D9663) for 1 h at room temperature (RT). Samples were incubated with primary mouse anti-human LC3B antibody (1:100, Cell Signaling Technology, Catalog number: 83506) over night at 4°C. Afterwards, samples were extensively washed in PBS and incubated for 2 h at RT in the dark with secondary antibody donkey anti-mouse AF488 (1:1000, Invitrogen, Catalog number: A-21202) and the nucleus counterstain 4′,6-Diamidino-2-phenyl-indol-dihydrochloride (DAPI 1:2000, Sigma-Aldrich, Catalog number: MBD0015). Samples were washed 3 times with PBS and semi-dry mounted with glass cover slips in ProLong Gold Antifade Mountant (Invitrogen, Catalog number: P36934). Cells were analyzed using a Zeiss Observer Z1 fluorescence microscope equipped with an Abberior Instruments STEDYCON unit for confocal and super-resolution STED microscopy. Representative confocal z-stacks were taken with a 100× objective. Images show maximum intensity projections and were post-processed with Fiji (ImageJ1.54f) and Microsoft PowerPoint.

**Statistical analysis**

Mice were randomly assigned to the experimental groups. No blinding was used for the injection or analysis. There were no exclusion criteria for animals. Statistical analyses were performed with GraphPad Prism 8 software (GraphPad Software, San Diego, CA, USA). Data was tested for normality and appropriate statistical tests were used: Statistical analyses were performed by Wilcoxon matched-pairs signed rank test, a paired or two-tailed unpaired t-test for the analysis between two groups, one-way ANOVA with Dunnett’s or Tukey’s post-hoc test for multiple comparisons, a two-way ANOVA with Tukey’s or Šídák's post-hoc test was performed for multiple comparisons and a Cox proportional hazards model was used for the statistical analysis of the Kaplan-Meier curve. Significance levels are defined as follows: *, p ≤ 0.05; **, p ≤ 0.01; ***, p ≤ 0.001; ****, p ≤ 0.0001; ns, not significant.

**References**

1. Ge SX, Jung D, Yao R. ShinyGO: a graphical gene-set enrichment tool for animals and plants. Bioinformatics. 2020;36(8):2628-9.

2. Haferlach T, Kohlmann A, Wieczorek L, Basso G, Kronnie GT, Bene MC, et al. Clinical utility of microarray-based gene expression profiling in the diagnosis and subclassification of leukemia: report from the International Microarray Innovations in Leukemia Study Group. J Clin Oncol. 2010;28(15):2529-37.

3. Kohlmann A, Kipps TJ, Rassenti LZ, Downing JR, Shurtleff SA, Mills KI, et al. An international standardization programme towards the application of gene expression profiling in routine leukaemia diagnostics: the Microarray Innovations in LEukemia study prephase. Br J Haematol. 2008;142(5):802-7.

4. Liu WM, Li R, Sun JZ, Wang J, Tsai J, Wen W, et al. PQN and DQN: algorithms for expression microarrays. J Theor Biol. 2006;243(2):273-8.

5. Bennett JM, Catovsky D, Daniel MT, Flandrin G, Galton DA, Gralnick HR, et al. Proposals for the classification of the acute leukaemias. French-American-British (FAB) co-operative group. Br J Haematol. 1976;33(4):451-8.

6. Metzeler KH, Hummel M, Bloomfield CD, Spiekermann K, Braess J, Sauerland MC, et al. An 86-probe-set gene-expression signature predicts survival in cytogenetically normal acute myeloid leukemia. Blood. 2008;112(10):4193-201.

7. Frauenlob T, Neuper T, Regl C, Schaepertoens V, Unger MS, Oswald AL, et al. Helicobacter pylori induces a novel form of innate immune memory via accumulation of NF-small ka, CyrillicB proteins. Front Immunol. 2023;14:1290833.

8. Sanjana NE, Shalem O, Zhang F. Improved vectors and genome-wide libraries for CRISPR screening. Nat Methods. 2014;11(8):783-4.

9. Doench JG, Fusi N, Sullender M, Hegde M, Vaimberg EW, Donovan KF, et al. Optimized sgRNA design to maximize activity and minimize off-target effects of CRISPR-Cas9. Nat Biotechnol. 2016;34(2):184-91.

10. Sanson KR, Hanna RE, Hegde M, Donovan KF, Strand C, Sullender ME, et al. Optimized libraries for CRISPR-Cas9 genetic screens with multiple modalities. Nat Commun. 2018;9(1):5416.

11. Kasper M, Regl G, Eichberger T, Frischauf AM, Aberger F. Efficient manipulation of Hedgehog/GLI signaling using retroviral expression systems. Methods Mol Biol. 2007;397:67-78.

12. Detre S, Saclani Jotti G, Dowsett M. A "quickscore" method for immunohistochemical semiquantitation: validation for oestrogen receptor in breast carcinomas. J Clin Pathol. 1995;48(9):876-8.

13. Neuper T, Ellwanger K, Schwarz H, Kufer TA, Duschl A, Horejs-Hoeck J. NOD1 modulates IL-10 signalling in human dendritic cells. Sci Rep. 2017;7(1):1005.

14. Cox J, Mann M. MaxQuant enables high peptide identification rates, individualized p.p.b.-range mass accuracies and proteome-wide protein quantification. Nat Biotechnol. 2008;26(12):1367-72.

15. UniProt C. UniProt: a worldwide hub of protein knowledge. Nucleic Acids Res. 2019;47(D1):D506-D15.

16. Stacklies W, Redestig H, Scholz M, Walther D, Selbig J. pcaMethods--a bioconductor package providing PCA methods for incomplete data. Bioinformatics. 2007;23(9):1164-7.

17. Willforss J, Chawade A, Levander F. NormalyzerDE: Online Tool for Improved Normalization of Omics Expression Data and High-Sensitivity Differential Expression Analysis. J Proteome Res. 2019;18(2):732-40.

18. Liberzon A, Birger C, Thorvaldsdottir H, Ghandi M, Mesirov JP, Tamayo P. The Molecular Signatures Database (MSigDB) hallmark gene set collection. Cell Syst. 2015;1(6):417-25.

19. Seal RL, Braschi B, Gray K, Jones TEM, Tweedie S, Haim-Vilmovsky L, et al. Genenames.org: the HGNC resources in 2023. Nucleic Acids Res. 2023;51(D1):D1003-D9.

20. Perez-Riverol Y, Csordas A, Bai J, Bernal-Llinares M, Hewapathirana S, Kundu DJ, et al. The PRIDE database and related tools and resources in 2019: improving support for quantification data. Nucleic Acids Res. 2019;47(D1):D442-D50.
